# Long-lasting redundant *gnrh1/3* expression in GnRH neurons enabled apparent switching of paralog usage during evolution

**DOI:** 10.1016/j.isci.2024.109304

**Published:** 2024-02-22

**Authors:** Chika Fujimori, Kohei Sugimoto, Mio Ishida, Christopher Yang, Daichi Kayo, Soma Tomihara, Kaori Sano, Yasuhisa Akazome, Yoshitaka Oka, Shinji Kanda

**Affiliations:** 1 Atmosphere and Ocean Research Institute, The University of Tokyo, Kashiwa, Chiba, Japan; 2 Optics and Imaging Facility, National Institute for Basic Biology, Okazaki, Aichi, Japan; 3 Department of Biological Sciences, Graduate School of Science, The University of Tokyo, Bunkyo-ku, Tokyo, Japan; 4 Department of Neuroscience, Johns Hopkins University, Baltimore, MD, USA; 5 Graduate School of Life Sciences, Tohoku University, Sendai, Miyagi, Japan; 6 Nagahama Institute of Bio-Science and Technology, Nagahama, Shiga, Japan; 7 Department of Chemistry, Faculty of Science, Josai University, Sakado, Saitama, Japan; 8 Department of Anatomy, St. Marianna University School of Medicine, Kawasaki, Kanagawa, Japan

**Keywords:** Cellular neuroscience, Evolutionary mechanisms, Evolutionary theories, Ichthyology, Phylogenetics

## Abstract

Expressed subtype of paralogous genes in functionally homologous cells sometimes show differences across species, the reasons for which have not been explained. The present study examined hypophysiotropic gonadotropin-releasing hormone (GnRH) neurons in vertebrates to investigate this mechanism. These neurons express either *gnrh1* or *gnrh3* paralogs, depending on the species, and apparent switching of the expressed paralogs in them occurred at least four times in vertebrate evolution. First, we found redundant expression of *gnrh1* and *gnrh3* in a single neuron in piranha and hypothesized that it may represent an ancestral GnRH system. Moreover, the *gnrh1/gnrh3* enhancer of piranha induced reporter RFP/GFP co-expression in a single hypophysiotropic GnRH neuron in both zebrafish and medaka, whose GnRH neurons only express either *gnrh3* or *gnrh1*. Thus, we propose that redundant expression of *gnrh1/3* of relatively recent common ancestors may be the key to apparent switching of the paralog usage among present-day species.

## Highlights

• Piranha have *gnrh1* and *gnrh3* co-expressing hypophysiotropic GnRH neurons
• Relatively recent common ancestors of teleosts may have co-expressed *gnrh1* and *gnrh3*
• This might lead to inconsistent usage of paralogs (*gnrh1* or *gnrh3)* in GnRH neurons

## Graphical abstract

**Figure:**
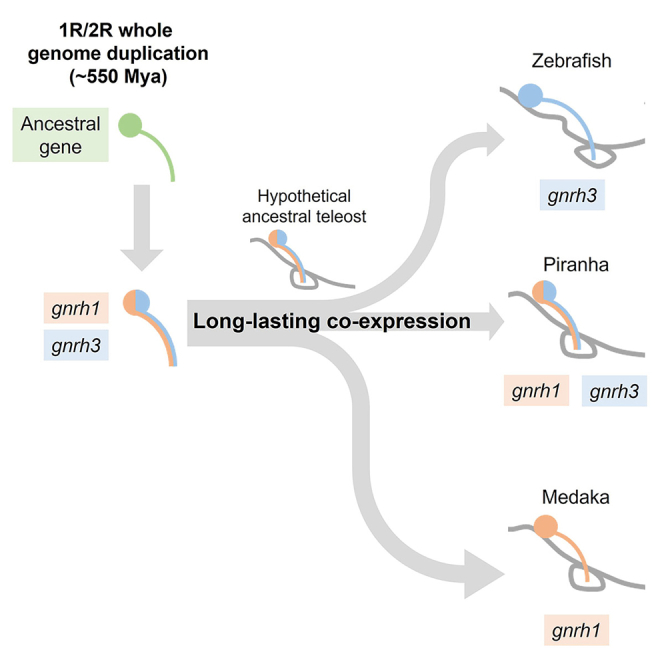

## Introduction

The theory of evolution by gene duplication tells us that after gene duplication, which is a main source of novel genes, one of the duplicated genes either degrades (non-functionalization) or acquires novel functions (neo-functionalization) while the other retains its original function, or both genes divide up the original function/expression pattern (sub-functionalization).^1^^,^^2^^,^^3^ In any case, duplicate genes lose their functional redundancy during evolution by accumulating mutations over a relatively short period of time,^3^^,^^4^^,^^5^ and once their fates have been fixed, their functional roles cannot be changed or swapped. Contrary to this principle, a strange phenomenon has been observed in a peptide hormone called gonadotropin-releasing hormone (GnRH), which is a key molecule for reproduction. In all vertebrate species that have been investigated thus far, one of the *gnrh* paralogous genes is expressed in GnRH neurons in the hypothalamus/preoptic area (POA), which project to the pituitary and control gonadal functions by inducing the release of gonadotropins (a so-called hypophysiotropic function). The functional subtype (paralog) of the *gnrh* gene for pituitary regulation has been shown to vary across species, and the expressed paralog has apparently switched several times during vertebrate evolution.^6^^,^^7^ There are three *gnrh* paralogs (*gnrh1/2/3*) which appeared after the first/second-round (1R/2R) whole genome duplication (WGD) ∼550 million years ago (Mya)^8^^,^^9^^,^^10^^,^^11^ (Figure S1). In many species including tetrapods and some teleosts, *gnrh1* is expressed in the hypophysiotropic GnRH neurons as the main regulator of gonadotropin release from the pituitary.^12^^,^^13^^,^^14^^,^^15^^,^^16^^,^^17^ However, some teleost species, including zebrafish, express *gnrh3* in hypophysiotropic GnRH neurons instead of *gnrh1*. Interestingly, all species that express *gnrh3* in hypophysiotropic GnRH neurons have genetically lost *gnrh1*^18^^,^^19^^,^^20^ (Figure S1). Since the peptide sequences among different GnRH paralogs are highly conserved, and their ligand-receptor relationship has been considered promiscuous (all the GnRH subtypes basically show similar actions on the GnRH receptors),^21^^,^^22^ *gnrh3* has been considered to have compensated for the loss of *gnrh1* during evolution.^6^^,^^7^ On the other hand, *gnrh2* is expressed in the midbrain in both teleosts and tetrapods.^8^ GnRH2-expressing neurons project to various brain areas but not to the pituitary,^12^ and are suggested not to be involved in gonadotropin release.^23^^,^^24^ Therefore, it appears that *gnrh3* is solely responsible for functional compensation for the loss of *gnrh1*. However, no study has yet provided a reasonable explanation as to why functionally homologous neurons express different paralogs among different species.

This switch in paralog usage in the hypophysiotropic GnRH neurons has occurred at least four times in the evolutionary lineage of teleosts (Figure S1). If we can find a species whose hypophysiotropic GnRH neurons co-express both *gnrh1* and *gnrh3*, this should help to explain the mechanism for this apparent switch in paralog expression among different species. More specifically, co-expression of *gnrh1* and *gnrh3* in a common ancestor’s hypophysiotropic GnRH neurons may allow downstream species to lose one of these paralogs. Interestingly, we found reports on *gnrh* paralog genes in two characiform species (order Characiformes) showing different situations. In pacu (*Piaractus mesopotamicus*, family Serrasalmidae), three different forms of GnRH peptides were identified by using HPLC analysis followed by mass spectrometry,^25^ whereas the *gnrh1* gene could not be identified in another Characiform family, Characidae species, including *Astyanax altiparanae*^26^ and Mexican tetra (Figure S1). Since it suggests that Characidae species lost *gnrh1* after diverged with Serrasalmidae, we surmised that species in the family of Serrasalmidae, as a presumed ancestral GnRH system, may provide a hint toward understanding the mechanism underlying switches in *gnrh* paralog expression.

In the present study, to understand the evolutionary mechanism of switches in paralogous-gene usage, we analyzed the *gnrh1* and *gnrh3* system of a Serrasalmidae fish, the red-bellied piranha (*Pygocentrus nattereri*). First, we found in piranha that *gnrh1* and *gnrh3* are co-expressed in hypophysiotropic GnRH neurons, which suggests that the hypophysiotropic GnRH neurons in a common ancestral teleost should have also expressed both paralogs. Therefore, we surmised that the co-expression of *gnrh* paralogs may explain the apparent switch in paralog usage in hypophysiotropic neurons: co-expression may have permitted the loss of either *gnrh1* or *gnrh3* expression/genes for regulating gonadotropin release without severe functional defects. Furthermore, to provide experimental support for this hypothesis, the enhancer activity of the piranha *gnrh* gene was tested in other fishes, medaka and zebrafish, which exhibit different *gnrh1/3* systems.

## Results

### In piranha, *gnrh1* and *gnrh3* are co-expressed in POA GnRH neurons

**Figure 1:**
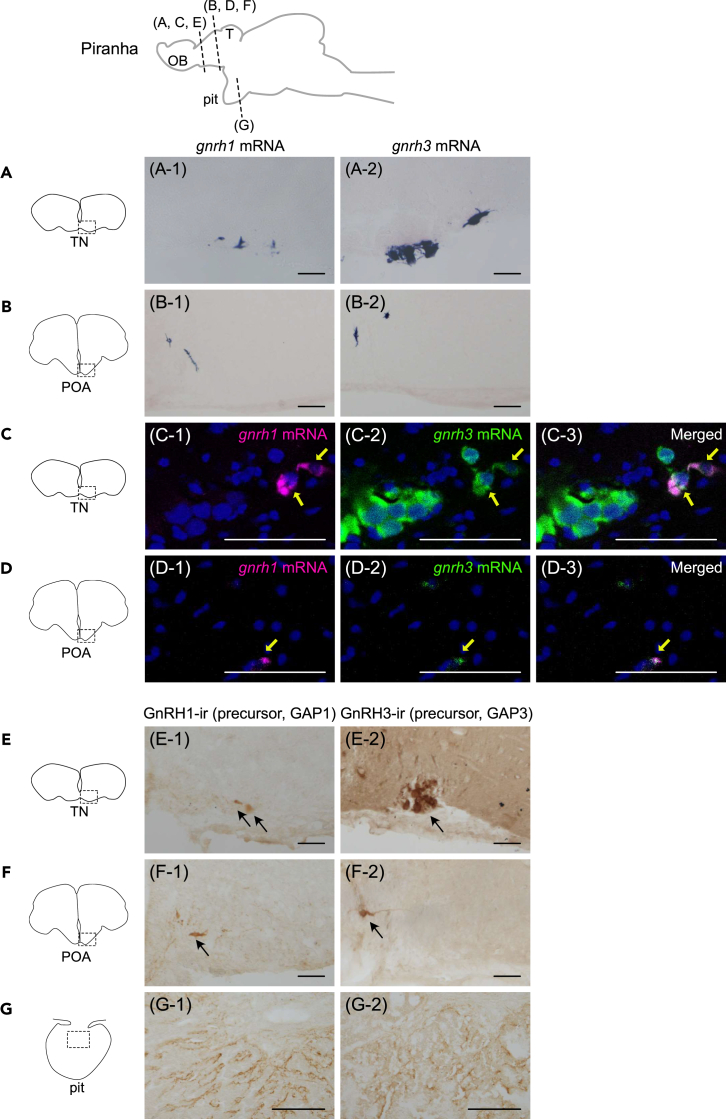
In piranha, *gnrh1* and *gnrh3* are co-expressed in POA GnRH neurons (A and B) Both *gnrh1* (A-1, B-1) and *gnrh3* (A-2, B-2) mRNA are expressed in the terminal nerve (TN) (A) and the preoptic area (POA) (B). (C and D) Double *in situ* hybridization indicates that *gnrh1* and *gnrh3* mRNA are co-expressed in the same neuron in the TN (C-1, *gnrh1* mRNA; C-2, *gnrh3* mRNA; C-3, merged) and the POA (D-1, *gnrh1* mRNA; D-2, *gnrh3* mRNA; D-3, merged). Yellow arrows indicate the cells that co-express *gnrh1* and *gnrh3* mRNA. Note that neuronal cluster with giant cell bodies only express *gnrh3*. (E and F) Immunohistochemistry using precursor of GnRH1 (E-1, F-1) or GnRH3 (E-2, F-2)-specific antibody labeled the cell bodies in the TN (E) and the POA (F), which is consistent with the localization indicated by *in situ* hybridization. Arrows indicate cell bodies. (G) Both GnRH1- (G-1) and GnRH3- (G-2) immunoreactive (ir) fibers are observed in the pituitary, which suggests the redundant regulation of LH release by GnRH1 and GnRH3 peptides. Nuclear counterstaining using methyl green is shown as blue signals. Scale bars represent 100 μm (A, B, E, F, G) and 50 μm (C, D), respectively. OB, olfactory bulb; T, telencephalon; pit, pituitary.

Sequences of piranha *gnrh1* and *gnrh3* mRNA were successfully determined by 5′ and 3′ RACE method (Figures S2A and S2B). Also, sequences of head-and-tail-light tetra (*Hemigrammus ocellifer*, Characidae) *gnrh3* mRNA were isolated by 3′ RACE followed by RT-PCR (Figure S2C). Analyses of alignment (Figures S2D and S2E) and the phylogenetic tree (Figure S3A) of precursor sequences strongly suggest that these genes are piranha *gnrh1*, piranha *gnrh3* and head-and-tail-light tetra *gnrh3*. *In situ* hybridization using piranha *gnrh1* and *gnrh3* specific probes demonstrated that both *gnrh1* and *gnrh3* mRNA-expressing neurons are localized in the terminal nerve (TN) as well as the POA in the piranha brain (Figures 1A and 1B). Since some *gnrh1*-expressing and *gnrh3*-expressing neurons appeared to be localized in adjacent areas of the brain, we performed double *in situ* hybridization of *gnrh1* and *gnrh3* to examine whether they are co-expressed in the same neuron. Double *in situ* hybridization clearly demonstrated that in the TN, larger cells forming a cluster (TN ganglion) only express *gnrh3* (a cluster of large green cells shown in the left part of Figure 1C) whereas a high percentage of smaller cells co-express both *gnrh1* and *gnrh3* (yellow arrows in Figure 1C). We also demonstrated that some neurons in POA co-expressed *gnrh1* and *gnrh3* mRNA (Figure 1D, yellow arrows). Note that because of the scattered distribution of *gnrh* mRNA-expressing cells in the POA, it was impossible to include more than one *gnrh* mRNA-expressing cell in a single section. Therefore, images of other sections were shown in the Figure S4.

### Both GnRH1-immunoreactive(ir) and GnRH3-ir neuronal fibers project to the pituitary in piranha

Using the newly generated antibodies against piranha GnRH1 or GnRH3 precursor for immunohistochemistry (GAP1/GAP3), we analyzed the axonal projection of GnRH1 and GnRH3 neurons in piranha after scrutinizing the specificities of these antibodies by pre-absorption with the peptides (Figure S5). Both the GnRH1-ir and GnRH3-ir cell bodies were localized in TN as well as POA (Figures 1E and 1F), and the densely labeled axonal fibers of both GnRH1-ir and GnRH3-ir neurons were observed in the pituitary (Figure 1G).

### *gnrh*1 knockout piranha show a similar pattern of GnRH innervation to that of head-and-tail-light tetra

**Figure 2:**
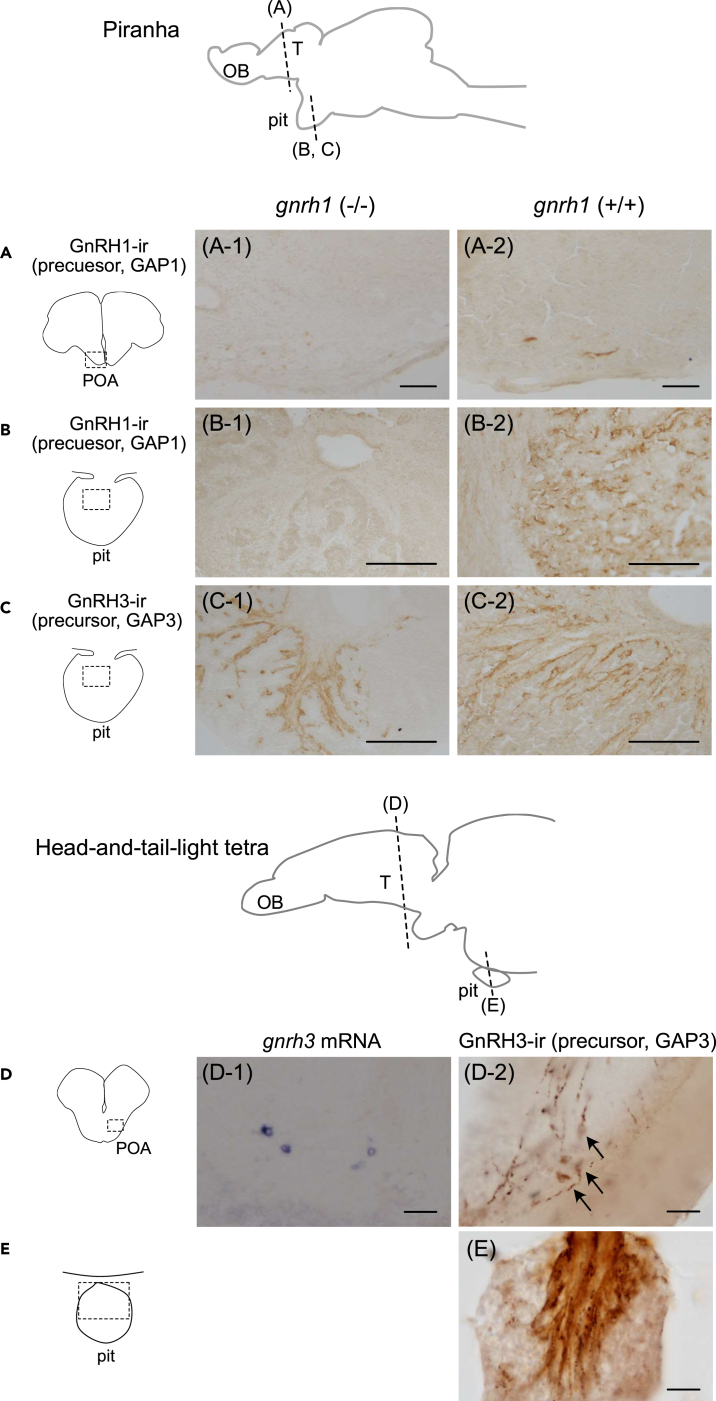
*gnrh1* knockout (KO) piranha show innervation of GnRH3-ir fibers in the pituitary, similar to other characiform fishes (A) GnRH1 precursor-ir cell bodies are not observed in the *gnrh1* KO piranha (A-1), while they are observed in the wild type (A-2). (B) GnRH1 precursor-ir fibers are not found in the pituitary of the *gnrh1* KO (B-1), while they are observed in the wild type (B-2). (C) GnRH3-ir fibers are observed in the pituitary of both *gnrh1* KO (C-1) and wild type (C-2) piranha. (D) In head-and-tail-light tetra, *in situ* hybridization (D-1) and immunohistochemistry (D-2) shows that *gnrh3/*GnRH3-expressing neurons are localized in the POA. (E) Immunohistochemistry indicates that GnRH3 precursor-ir axonal projection is observed in the pituitary. Arrows indicate cell bodies. Scale bars represent 100 μm (A-C) and 20 μm (D, E), respectively. OB, olfactory bulb; T, telencephalon; pit, pituitary.

Next, to investigate the functional compensation for the loss of *gnrh1* by the *gnrh3* gene, which purportedly occurred in Characidae lineages (Figure S1), we established a new artificial fertilization method and generated *gnrh1* knockout (KO) piranha using CRISPR/Cas9. After microinjection of the CRISPR/Cas9 mixture, F0 embryos were raised and incrossed to generate the F1 generation. In the F1 generation, PCR and subsequent sequencing analysis indicated that there were individuals that had frameshift mutation and resulted in non-functional GnRH peptide in the *gnrh1* gene (Figure S3B), which we expected would fail to produce functional GnRH1 peptides. Immunohistochemistry for piranha GAP1 showed that neither GnRH1-ir cell bodies in the POA nor fibers in the pituitary were observed in *gnrh1*^*−/−*^ piranha (Figures 2A-1 and 2B-1), whereas both were observed in *gnrh1*^*+/+*^ piranha (Figures 2A-2 and B-2). In both *gnrh1*^*−/−*^ and *gnrh1*^*+/+*^ piranha, GnRH3-ir fibers were similarly observed in the pituitary (Figures 2C-1 and C-2). This result of *gnrh1* KO piranha, which possess only *gnrh3* in hypophysiotropic GnRH neurons, was similar to the head-and-tail-light tetra, a closely related species that does not possess the *gnrh1* gene. In fact, in the POA of head-and-tail-light tetra, *gnrh3* was detected in the cell bodies of POA by *in situ* hybridization (Figure 2D-1) as well as immunohistochemistry (Figure 2D-2), and GnRH3-ir fibers were observed in the pituitary (Figure 2E).

### In zebrafish, piranha *gnrh1* and *gnrh3* enhancers can be activated in the POA GnRH neurons expressing *gnrh3* mRNA

**Figure 3:**
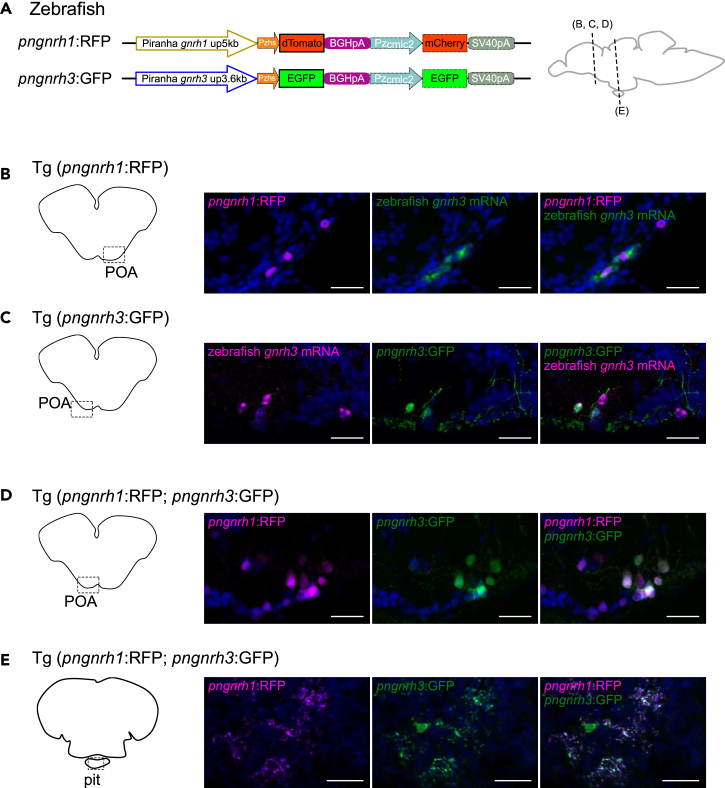
In zebrafish, piranha *gnrh1* and *gnrh3* enhancers can be activated in the POA GnRH neurons expressing *gnrh3* mRNA (A) The constructs used to generate transgenic zebrafish. Both constructs examine the enhancer activity of piranha *gnrh1* or *gnrh3* 5′flanking region by using basal promoter (zebrafish heat shock promoter, Pzhs) and a fluorescent protein (RFP/dTomato or GFP/EGFP). For screening of embryos, cardiac myosin light chain 2 promoter of zebrafish (Pzcmlc2), mCherry or EGFP and SV40 poly(A) signal were inserted downstream of the reporter construct. (B and C) Double labeling of piranha enhancer-induced fluorescent proteins and the intrinsic mRNA of zebrafish. (B) In Tg (*pngnrh1*:RFP) zebrafish, *pngnrh1* enhancer-induced RFP expression is observed in the GnRH3 neurons (*gnrh3* mRNA-expressing neurons) in the POA. (C) In Tg (*pngnrh3*:GFP) zebrafish, *pngnrh3* enhancer-induced GFP expression is observed in the GnRH3 neurons in the POA. (D and E) Analysis of the double transgenic zebrafish, Tg (*pngnrh1*:RFP; *pngnrh3*:GFP). (D) In Tg (*pngnrh1*:RFP; *pngnrh3*:GFP) zebrafish, some of the neurons in the POA express both RFP and GFP, suggesting that *pngnrh1* and *pngnrh3* enhancers are active in the same neurons. (E) In the pituitary of Tg (*pngnrh1*:RFP; *pngnrh3*:GFP) zebrafish, neuronal fibers that are labeled by both RFP and GFP are observed, suggesting that the RFP and GFP co-expressing neurons in the POA are hypophysiotropic. Nuclear counterstaining using methyl green is shown as blue signals. Scale bars, 20 μm.

The fact that *gnrh1* and *gnrh3* were co-expressed in hypophysiotropic GnRH neurons in piranha strongly suggests that the common ancestor of Ostariophysi possessed hypophysiotropic GnRH neurons expressing both *gnrh1* and *gnrh3*. Given this ancestor, the GnRH system in the present ostariophysian fishes, in which either *gnrh1* or *gnrh3* are expressed in the hypophysiotropic GnRH neurons, can be explained to have arisen as a consequence of a simple loss of either one of the two *gnrh* paralogs. We thus examined whether the *trans-*regulatory elements in zebrafish hypophysiotropic GnRH neurons can still activate the anciently lost *gnrh1* enhancer. We generated transgenic (Tg) zebrafish harboring RFP (dTomato) or GFP (EGFP) as reporter genes under the regulation of piranha *gnrh1* or *gnrh3* 5′ flanking regions, Tg (*pngnrh1*:RFP) and Tg (*pngnrh3*:GFP), respectively (Figures 3A, S6, and S7). In the established transgenic zebrafish, both piranha *gnrh1* (RFP) and *gnrh3* (GFP) transcriptional activity was observed in the cell bodies of endogenous GnRH3 neurons in the POA (Figures 3B and 3C), and reporter expression of RFP and GFP was observed in 42–65% and 53–55% of endogenous *gnrh3*-expressing cells, respectively. These results indicate that piranha *gnrh1* and *gnrh3* enhancers were activated in zebrafish GnRH neurons. These results strongly suggest that *trans-*regulatory elements activating both *gnrh1* and *gnrh3* enhancers are conserved, even though the *gnrh1* gene has already been lost in zebrafish. Furthermore, in the double transgenic zebrafish, Tg (*pngnrh1*:RFP; *pngnrh3*:GFP), RFP and GFP expression was co-localized in both cell bodies in the POA (Figure 3D) and the axons in the pituitary (Figure 3E).

### In medaka, both piranha *gnrh1* and *gnrh3* enhancers can be activated in POA GnRH neurons expressing intrinsic *gnrh1* mRNA

**Figure 4:**
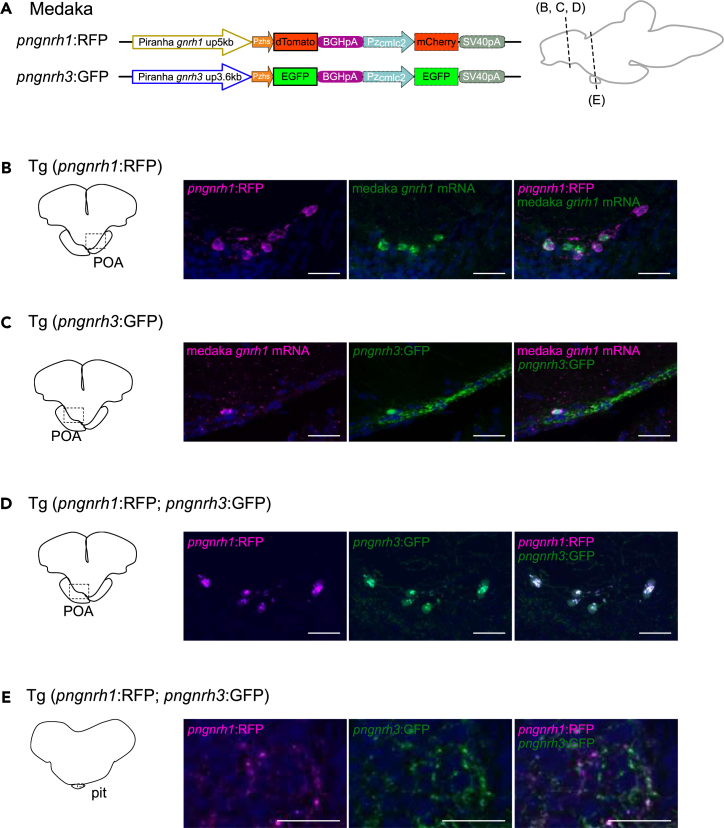
In medaka, both piranha *gnrh1* and *gnrh3* enhancers can be activated in POA GnRH neurons expressing intrinsic *gnrh1* mRNA (A) The constructs used to generate transgenic medaka. Both constructs examine the enhancer activity of piranha *gnrh1* or *gnrh3* 5′flanking region by using a basal promoter (zebrafish heat shock promoter, Pzhs) and a fluorescent protein (RFP/dTomato or GFP/EGFP). For screening of embryos, cardiac myosin light chain 2 promoter of zebrafish (Pzcmlc2), mCherry or EGFP and SV40 poly(A) signal were inserted downstream of the reporter construct. (B and C) Double labeling of piranha enhancer-induced fluorescent proteins and the intrinsic mRNA of medaka. (B) In Tg (*pngnrh1*:RFP) medaka, *pngnrh1* enhancer-induced RFP expression is observed in the GnRH1 neurons (*gnrh1* mRNA-expressing neurons) in the POA. (C) In Tg (*pngnrh3*:GFP) medaka, *pngnrh3* enhancer-induced GFP expression is also observed in the GnRH1 neurons in the POA. (D and E) Analysis of the double transgenic medaka, Tg (*pngnrh1*:RFP; *pngnrh3*:GFP). (D) In Tg (*pngnrh1*:RFP; *pngnrh3*:GFP) medaka, some of the neurons in the POA expressed both RFP and GFP suggesting that *pngnrh1* and *pngnrh3* enhancers are active in the same neurons. (E) In the pituitary of Tg (*pngnrh1*:RFP; *pngnrh3*:GFP) medaka, neuronal fibers that are labeled by both RFP and GFP are observed, which suggests that the RFP and GFP co-expressing neurons in the POA are hypophysiotropic neurons. Nuclear counterstaining using methyl green is shown as blue signals. Scale bars, 20 μm.

Although both *gnrh1* and *gnrh3* genes are retained in all acanthopterygian fishes examined thus far including medaka, hypophysiotropic GnRH neurons express *gnrh1* but not *gnrh3* in principle (Figure S1).^12^^,^^13^^,^^15^^,^^17^ To examine whether the lack of expression of *gnrh3* in hypophysiotropic GnRH neurons in Acanthopterygii was due to changes in the enhancer sequence, we generated transgenic medaka, Tg (*pngnrh1*:RFP) and Tg (*pngnrh3*:GFP) and examined the enhancer activity of piranha *gnrh1* and *gnrh3* enhancers, which are hypothetical ancestral enhancers, in the hypophysiotropic neurons of medaka (Figure 4A). In transgenic medaka, both piranha *gnrh1* and *gnrh3* enhancers induced RFP (dTomato)/GFP (EGFP) reporter expression in medaka *gnrh1* mRNA-expressing cell bodies in the POA (Figures 4B and 4C), and 88–96% and 10–22% of hypophysiotropic *gnrh1* neurons in the POA expressed RFP (piranha *gnrh1*) and GFP (piranha *gnrh3*), respectively. Moreover, in the double transgenic medaka, Tg (*pngnrh1*:RFP; *pngnrh3*:GFP), RFP and GFP expression was co-localized in both cell bodies in the POA (Figure 4D) and the axons in the pituitary (Figure 4E).

## Discussion

Although many comparative anatomical studies have reported inconsistencies among species as to which paralog, *gnrh1* or *gnrh3,* is expressed in hypophysiotropic GnRH neurons, the reason for this inconsistency has been unknown. In the present study, we found that both *gnrh1* and *gnrh3* are co-expressed in hypophysiotropic neurons in piranha. The duplication of *gnrh1* and *gnrh3* genes occurred in the 1R/2R WGD, which implies that both of them should have been expressed in the same cell just after the gene duplication event because they possessed identically copied *cis-*regulatory sequences. Given that *gnrh1* and/or *gnrh3* expressing neurons are only a few dozen among the uncountable number of neurons in the POA, the most parsimonious interpretation of redundant regulation of piranha hypophysiotropic GnRH neurons is that the *gnrh1* and *gnrh3* co-expression pattern has been inherited since the 1R/2R WGD, rather than assuming that a coincidental reunion occurred after their expression had been differentiated. Furthermore, we analyzed the enhancer activities of piranha *gnrh1* and *gnrh3* in zebrafish, which have lost the *gnrh1* gene during evolution, and medaka, in which *gnrh3* is not expressed in hypophysiotropic GnRH neurons. The results revealed that both piranha *gnrh1* and *gnrh3* enhancers can be activated in hypophysiotropic GnRH neurons in medaka and zebrafish by their intrinsic *trans-*regulatory elements. This finding supports a hypothesis that the frequent switching of *gnrh* paralog usage in hypophysiotropic GnRH neurons during teleost evolution is due to the ancestral co-expression of *gnrh1* and *gnrh3* in hypophysiotropic neurons, which is still inherited by piranha. Thus, the analysis of these slowly evolving paralogous genes at the cellular level, including their expression as well as enhancer activities across species, provides a valuable model for understanding the mechanism of allocating distinctive roles to paralogs after gene duplication. The present approach also provides important insights into the evolutionary process of role-division in paralogs.

### Redundant expression of *gnrh1* and *gnrh3* in hypophysiotropic GnRH neurons in the hypothetical common ancestors may have played a permissive role in apparent switch of GnRH paralog usage responsible for gonadotropin release

The present study demonstrated that piranha possess both *gnrh1* and *gnrh3* genes and show redundant expression of *gnrh1* and *gnrh3* genes in hypophysiotropic GnRH neurons. We also showed that smaller cells, but not large cells, in the TN express both *gnrh1* and *gnrh3* genes. It has been generally accepted that all GnRH neurons in the forebrain (including hypophysiotropic GnRH neuron) originate from the olfactory placodes and migrate to their destination. The large GnRH cells stop migrating in the rostral forebrain to form the TN ganglion. On the other hand, smaller GnRH neurons, which include hypophysiotropic GnRH neurons, are sparsely distributed along the migratory pathway from the olfactory bulb to POA.^17^^,^^27^ Therefore, the smaller cells in the TN can be considered to have the developmental origin similar to the hypophysiotropic GnRH neurons in the POA, despite their location. It thus follows that a considerable percentage of cells expressing both *gnrh1* and *gnrh3* are observed in smaller GnRH neurons in the TN, while the large TN ganglion cells only express *gnrh3*.

Also, both GnRH1-ir and GnRH3-ir fibers were abundantly observed in the pituitary (Figure 1G), which are suggested to originate from POA *gnrh1* and *gnrh3* co-expressing neurons (Figure 1D). In many vertebrates, it is observed that the number of GnRH neuron axons comprising the hypophysiotropic projection is large compared to the small number of cell bodies in the POA.^14^^,^^28^^,^^29^ Therefore, it is also reasonable that a rather small number of GnRH neurons heavily innervate the pituitary in piranha.

On the other hand, closely related Characiform/Characidae species examined to date were suggested to have lost *gnrh1*, based on analysis of genome database of Mexican tetra and RT-PCR results of neon tetra, head-and-tail-light tetra, and glowlight tetra (Figure S3C). Taken together, both *gnrh1* and *gnrh3* can be considered to have been conserved from the time of emergence of Characiformes (125 Mya). Later, Serrasalmidae species (e.g., piranha and pacu) conserved both paralogs, whereas Characidae (e.g., neon tetra, Mexican tetra) lost *gnrh1* during early evolutionary stages. Many other species that have been examined in Ostariophysi have lost either *gnrh3* (all species examined in Siluriform) or *gnrh1* (all species examined in Cypriniform),^20^^,^^30^^,^^31^^,^^32^^,^^33^^,^^34^ which indicates that they lost either *gnrh1* or *gnrh3* independently from the ancestor that co-expresses *gnrh1* and *gnrh3* in hypophysiotropic neurons, like the piranha observed in the present study. The existence of such dual-paralog co-expressing ancestors likely caused the difference in expression of *gnrh* paralogs in hypophysiotropic GnRH neurons in the present-day species.^6^^,^^7^^,^^35^ This hypothesis is supported by the expression analysis of *gnrh1* KO piranha and head-and-tail-light tetra in the present study. Due to the inability to control piranha spawning, the fertility of the *gnrh1* KO piranha could not be estimated. Given that the release of GnRH and subsequent LH secretion on teleost ovulation is transient,^16^ proving the organisms’ inability to spawn requires an artificial method to fully control the timing the ovulation. As there is currently no established method to induce natural spawning of piranha, the function of *gnrh1* at the individual level cannot be assessed. However, we can speculate that *gnrh1* KO piranha can reproduce because they show dense GnRH3 fibers in the pituitary (Figure 2C-1), which should be able to regulate gonadotropin release as in other Characiform species that have lost *gnrh1*^26^ (e.g., all Characidae species examined in the present study). Recent reports have shown that early divergent species such as catsharks and coelacanths have both *gnrh1* and *gnrh3*.^9^^,^^11^ Future double labeling studies in these species may provide results that support the expression pattern of a common ancestor.

### Both piranha *gnrh1* and *gnrh3* enhancers can be activated in POA GnRH neurons expressing *gnrh3* mRNA in zebrafish, although they do not possess an intrinsic *gnrh1* gene

Given that piranha hypophysiotropic GnRH neurons co-express *gnrh1* and *gnrh3*, the common ancestor of Ostariophysi is likely to have possessed hypophysiotropic neurons that co-express *gnrh1* and *gnrh3*. According to this hypothesis, the present ostariophysian fish that lack the *gnrh1* gene may be able to activate gene expression via the *gnrh1* enhancer of piranha in their hypophysiotropic GnRH neurons. By using zebrafish as a model, we examined enhancer activity of piranha *gnrh1* and *gnrh3* in intrinsic *gnrh3* mRNA-expressing neurons of ostariophysian POA. This examination of heterologous enhancer activities, which has been widely used in evo-devo studies,^36^^,^^37^ provided suggestions for the evolution of the *gnrh* system in adult neurons in the present study.

**Figure 5:**
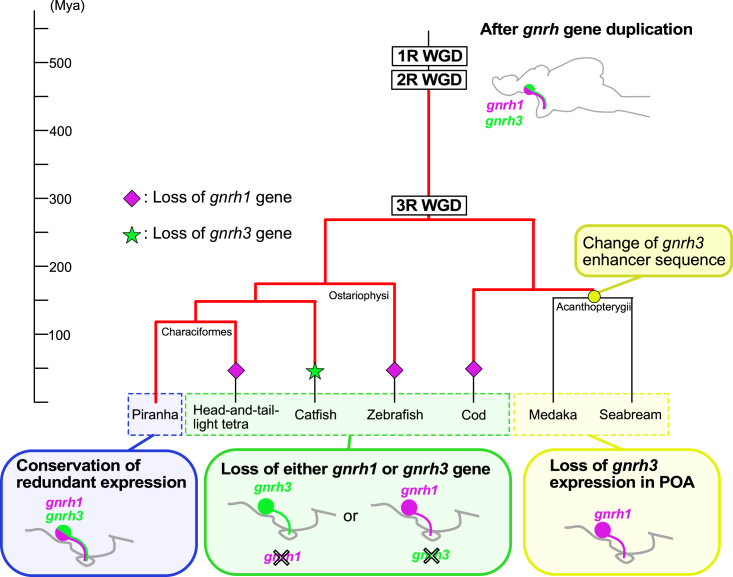
Working hypothesis of the evolution of paralogous *gnrh1/gnrh3* genes underlying the frequent switching of the *gnrh* gene expression in hypophysiotropic GnRH neurons The present study provides evidence that piranha *gnrh1* and *gnrh3* are co-expressed in the hypophysiotropic GnRH neurons (blue box), which suggests that all its ancestors inherited the same property since the 1R/2R WGD. This evidence is the key to explaining why the loss of either *gnrh1* or *gnrh3* gene have been permitted in many ancestral teleosts. The red lines indicate hypothetical ancestors that co-expressed *gnrh1* and *gnrh3* in the hypophysiotropic GnRH neurons. Many other species so far examined in Ostariophysi lost either *gnrh3* or *gnrh1* (green box). Unlike other orders, in Acanthopterygii, the POA neuron-specific enhancer of *gnrh3* is suggested to have been lost in their ancestors (yellow circle), which is consistent with the experimental evidence that *gnrh1* is used in hypophysiotropic neurons in all species examined in Acanthopterygii (yellow box). Purple diamond and green star indicate loss of *gnrh1* and *gnrh3*, respectively.

We demonstrated that both Tg (*pngnrh1*:RFP) and Tg (*pngnrh3*:GFP) zebrafish showed not only GFP (*gnrh3* enhancer induced) but also RFP (*gnrh1* enhancer induced) expression in the *gnrh3* mRNA-expressing hypophysiotropic neurons in the POA (Figure 3), even though zebrafish has lost the *gnrh1* gene, likely in the common ancestor of Cyprinidae and Danionidae (75 Mya)^33^ (Figure 5).

This result not only supports the hypothesis that *gnrh1* and *gnrh3* were co-expressed in hypophysiotropic GnRH neuron of their ancestor but also strongly suggests that transcription factors that can activate *gnrh1* enhancer have been conserved for an extended period of time in the absence of *gnrh1*. Given that *gnrh1* and *gnrh3* should have been transcribed by the same transcription factors at the time of duplication (1R/2R WGD), it is likely in zebrafish POA GnRH neurons that the transcription factors for intrinsic *gnrh3* could activate the piranha *gnrh1* enhancer. Although we could not specify the enhancer sequences because of low similarity in the 5′-flanking region sequence of *gnrh1* and *gnrh3*, a previous developmental study showing that the enhancers of paralogous genes were active in the same tissue^38^ supports the hypothesis that *gnrh1* and *gnrh3* have possessed common *cis-* and *trans-*expression regulatory systems. *In silico* analyses for transcription factors that could bind to the upstream sequences of piranha *gnrh1* and *gnrh3* revealed transcription factors that can bind to both sequences (Table S2). Some of these factors can be involved in transcription of *gnrh* paralogs in hypophysiotropic neuron in POA. Future studies may reveal their common transcriptional regulatory system.

### Both piranha *gnrh1* and *gnrh3* enhancers can be activated in hypophysiotropic POA GnRH neurons of medaka expressing intrinsic *gnrh1* mRNA

To uncover why most Acanthopterygii do not have hypophysiotropic *gnrh3* neurons in the POA despite possessing *gnrh3* gene itself,^12^^,^^13^^,^^15^^,^^17^ we generated transgenic medaka to examine whether medaka POA GnRH neurons are capable of activating gene expression via piranha *gnrh1* and *gnrh3* enhancers. Both Tg (*pngnrh1*:RFP) and Tg (*pngnrh3*:GFP) medaka showed enhancer activities of piranha *gnrh1* and *gnrh3* in POA *gnrh1* mRNA-expressing neurons (Figure 4), although medaka do not express their intrinsic *gnrh3* mRNA in hypophysiotropic POA neurons.^17^ The fact that the piranha *gnrh3* enhancer was activated in *gnrh1* mRNA-expressing neurons in POA of medaka indicates that medaka GnRH1 neurons in the POA possess a transcription factor that can activate the piranha *gnrh3* enhancer (Figure 4C). In other words, the loss of the POA-specific enhancer of the *gnrh3* gene in a common ancestor can explain why only *gnrh1* is expressed in hypophysiotropic neurons in acanthopterygians including medaka. Interestingly, although all present-day species in Acanthopterygii possess both *gnrh1* and *gnrh3*,^8^^,^^20^ *gnrh1* is exclusively expressed in the POA hypophysiotropic GnRH neurons in many species.^12^^,^^13^^,^^14^^,^^15^^,^^16^^,^^17^ Moreover, as *gnrh1* KO medaka are infertile due to ovulatory dysfunction,^39^ *gnrh1* is exclusively important for luteinizing hormone (LH) regulation.^40^ Given that *gnrh3* is seldom expressed in the POA of Acanthopterygii, the loss of the enhancer responsible for *gnrh3* expression in POA neurons may have occurred in the ancestor of medaka in the early acanthopterygian lineage (∼150 Mya), which forced all acanthopterygian to use their remaining *gnrh1* in the hypophysiotropic GnRH neurons in the POA. On the other hand, non-acanthopterygian Euteleostei, Atlantic cod^41^ have lost *gnrh1*, which indicates that the loss of the POA-specific enhancer of the *gnrh3* gene occurred at least after the divergence of Atlantic cod and Acanthopterygii (∼160 Mya) (Figure 5). Although the data in the present study suggested the homology of POA neurons of medaka *gnrh1*, zebrafish *gnrh3* and piranha *gnrh1/gnrh3* in terms of enhancer activity, it should be noted that the proportion of co-expressing reporter gene and endogenous *gnrh1* mRNA was higher in POA of Tg (*pngnrh1*:RFP) medaka (88–96%) than that of Tg (*pngnrh3*:GFP) medaka (10–22%). These results may imply that the common ancestor of medaka experienced not only the loss of the *gnrh3* enhancer sequence but also a partial change in the *trans-*regulatory system such as transcription factors.

In the present study, the enhancer activity of piranha *gnrh1* and *gnrh3* was analyzed in zebrafish/medaka TN neurons, in addition to the hypophysiotropic neurons. The enhancers of piranha *gnrh1* and *gnrh3* were also activated in *gnrh3* mRNA-expressing TN neurons in zebrafish (Figures S8B and S8C). Also, the double transgenic zebrafish Tg (*pngnrh1*:RFP; *pngnrh3*:GFP) showed co-localization of RFP and GFP in some cells in the TN (Figure S8D). This strongly suggests that zebrafish TN neurons have transcription factors to co-express *gnrh1* and *gnrh3* if they had an ancestral *gnrh1* gene. On the other hand, in medaka, reporter expression by the piranha *gnrh1* enhancer was not observed in TN *gnrh3* mRNA-expressing cells (Figure S8E), while *pngnrh3*:GFP reporter expression was observed (Figures S8F and S8G). In this case, we cannot distinguish whether these neurons do not have transcription factors to activate the piranha *gnrh1* enhancer or an incompatibility between medaka transcription factors and the piranha enhancer of this system prevents the transcription system.

On the other hand, all other cases in the POA neurons demonstrated the piranha’s enhancer activity in medaka or zebrafish neurons. These results clearly lead to the simple conclusion that the piranha *gnrh1/3* enhancer is active in zebrafish TN, POA, and medaka POA GnRH neurons, even though these species are phylogenetically distinct.

### The reason for prolonged conservation of redundant *gnrh1* and *gnrh3* expression after 1R/2R WGD

The present study revealed redundant co-expression of *gnrh1* and *gnrh3* in piranha hypophysiotropic POA GnRH neurons. Given that *gnrh1* and *gnrh3* arose in 1R/2R WGD, this implies that this co-expression pattern has been inherited by every ancestor of piranha for ∼550 million years. This long-lasting redundancy is surprising because redundant genes are generally eliminated immediately.^3^^,^^42^

According to simple gene dosage effects, an increase in gene copies affects the amount of gene expression.^43^^,^^44^ In fact, adaptive increases in copy number have been reported in some genes during evolution.^45^^,^^46^ Similarly, in *gnrh* genes expressed in a hypophysiotropic neuron, this redundancy of *gnrh1* and *gnrh3* should be conserved under positive selective pressure, since the amount of the gene product directly affects the efficiency of ovulation and the number of offspring. Interestingly, recently discovered hypophysiotropic neurons that have been proposed to stimulate FSH release exhibit a parallel scenario. In them, the neurotransmitters cholecystokinin a and cholecystokinin b, which arose in the 3R WGD, are co-expressed in medaka likely for the same reasons as GnRH1/3 neurons in piranha.^47^ It is possible that hypophysiotropic hormones that directly contribute to reproduction may tend to show such dosage effects. Furthermore, GnRH is suggested to regulate other hormones such as growth hormone and prolactin,^48^^,^^49^^,^^50^ which might be related to the redundancy. Further examination to investigate the relationship between such multifunctionality and the conservation of redundant *gnrh* paralog expression may be also intriguing.

However, the loss of *gnrh1* and *gnrh3* has occurred occasionally in the teleost lineage. These events suggest that the loss of either *gnrh1* or *gnrh3* did not have a large impact on survival, although it may be weakly deleterious. According to a theory in population genetics, natural selection does not theoretically work when the population size is small,^51^ and a weakly deleterious mutation may be fixed within a new population, also referred to as the founder effect.^52^ For these reasons, loss of either the *gnrh1* or *gnrh3* gene/enhancer may have occurred very slowly during the ∼550 My long history of vertebrate lineage, and the extreme case may be piranha, which conserves this redundancy even now (Figure 5).

### Slowly progressing role-division of paralogous genes *gnrh1* and *gnrh3* provides a good model for understanding paralogous gene evolution

The findings of *gnrh* genes demonstrated in the present study provide an intriguing example of the possible process of role-division of paralogous genes after duplication at the cellular level. Once genes are duplicated, they undergo neo-functionalization, sub-functionalization, or non-functionalization, causing their resulting paralogs to diverge into independent evolutionary paths, which usually prevents them from reuniting or swapping their roles. Unlike many genes that have undergone this process rapidly,^3^ *gnrh1*/*gnrh3* genes expressed in the hypophysiotropic GnRH neurons in vertebrates experienced this role-division process very slowly probably due to the weakly deleterious nature of their loss, which resulted in variation in paralog usage during evolution. Also, GnRH may not be the only example of paralogous gene switching,^53^ and further cellular-level observation of paralogous genes across species may further inform a general rule regarding the speed of role-division during evolution. The present cellular-level study explains the mechanism of the evolutionary process of role-division of very slowly evolving paralogous genes in hypophysiotropic neurons. These findings provide compelling evidence to support genomics-based theories on paralogous genes and offer a deeper understanding of their evolution.

### Limitations of the study

Based on the findings of histological study of piranha and examination of enhancer activities in zebrafish and medaka, this study suggested the common ancestor that allowed complicated situation of GnRH neurons in present day species. However, similar to many evolutionary studies, the overall hypothesis is established based on a hypothetical ancestor, and we did not perform experiments using a real common ancestor. Also, the effects of *gnrh1* KO in piranha was not physiologically examined and was only presumed by the axonal projection and the fertility of their close relatives naturally lost *gnrh1*.

## STAR★Methods

### Key resources table

**Table:** 

REAGENT or RESOURCE	SOURCE	IDENTIFIER
**Antibodies**		

Piranha Gnrh1 rabbit polyclonal antibodies	This paper (outsourced to Sigma-Aldrich)	N/A
Piranha Gnrh3 rabbit polyclonal antibodies	This paper (outsourced to Sigma-Aldrich)	N/A
Anti-RFP antibody	Rockland Immunochemicals	Cat#: 600-401-379S; RRID:AB_11182807
Anti-RFP antibody	Thermo Fisher Scientific	Cat# MA5-15257; RRID:AB_10999796
Anti-GFP rabbit IgG	Thermo Fisher Scientific	Cat#: A11120; RRID: AB_221569
Biotinylated goat anti-rabbit IgG	Vector Laboratories	Cat#: BA-1000; RRID: AB_2313606
Alkaline phosphatase-conjugated anti-DIG antibody	Roche Diagnostics	Cat#: 11093274910; RRID: AB_514497
Horseradish peroxidase-conjugated anti-FL antibody	Roche Diagnostics	Cat#: 11426346910; RRID:AB_840257
Horseradish peroxidase-conjugated anti-DIG antibody	Roche Diagnostics	Cat#: 11207733910; RRID: AB_514500
Alexa Fluor 555 conjugated anti-mouse IgG	Thermo Fisher Scientific	Cat#: A21422; RRID: AB_2535844

**Chemicals, peptides, and recombinant proteins**		

ISOGEN	Nippon gene	Cat#: 319-90211
SMART RACE kit	Takara Bio USA	Cat#: 634914
GenomeWalker Universal Kit	Takara Bio USA	Cat#: 638904
Tricaine methane sulfonate/ethyl 3-aminobenzoate methanesulfonate	Sigma-Aldrich	Cat#: E10521
Paraformaldehyde	Nakarai tesque	Cat#: 26126-25
Agarose, Type IX-A, Ultra-low Gelling Temperature	Sigma-Aldrich	Cat#: A2576-25G
Digoxigenin RNA Labeling Mix	Roche Diagnostics	Cat#: 11277073910
Fluorescein RNA Labeling Mix	Roche Diagnostics	Cat#: 11685619910
Proteinase K	TaKaRa Bio	Cat#: 9034
Phosphate-buffered saline Tablets	TaKaRa Bio	Cat#: T9181
Triton X-100	FUJIFILM Wako Pure Chemical Corporation	Cat#: 168-11805
50x Denharldt’s solution	FUJIFILM Wako Pure Chemical Corporation	Cat#: 043-21871
Calf Thymus DNA	Worthington Biochemical Corporation	Cat#: LS002105
tRNA	Roche Diagnostics	Cat#: 10109517001
Tris-buffered saline with Tween 20 Tablets	TaKaRa Bio	Cat#: T9141
T7 RNA polymerase	Roche Diagnostics	Cat#: 10881775001
SP6 RNA polymerase	Roche Diagnostics	Cat#: 10810274001
4-Nitro blue tetrazolium chloride (NBT)	Sigma-Aldrich	Cat#: N6639-250MG
5-Bromo-4-chloro-3-indolyl phosphate (BCIP)	Sigma-Aldrich	Cat#: B8503-100MG
SIGMAFAST™ Fast Red TR/Naphthol tablet	Sigma-Aldrich	Cat#: F4648
Tyramide Signal Amplification/Plus Cyanine3	Perkin Elmer	Cat#: NEL763B001KT
VECTASTAIN Elite ABC kit	Vector Laboratories	Cat#: PK-6100
Streptavidin, Alexa Fluor 488 conjugate	Thermo Fisher Scientific	Cat#: S11223
Streptavidin, Alexa Fluor 555 conjugate	Thermo Fisher Scientific	Cat#: S21381
Alexa Fluor 488 Tyramide SuperBoost Kit	Thermo Fisher Scientific	Cat#: B40922
Methyl green	FUJIFILM Wako Pure Chemical Corporation	Cat#: 138-12701
CC/Mount	Sigma-Aldrich	Cat#: C9368

**Deposited data**		

Piranha *gnrh1*	This paper	LC786488
Piranha *gnrh3*	This paper	LC786489
Head-and-tail-light tetra *gnrh3*	This paper	LC785397
Medaka *gnrh1*	NCBI	NM_001104699
Medaka *gnrh3*	NCBI	NM_001104672
Zebrafish *gnrh3*	Ensembl genome browser	ENSDARG00000056214

**Experimental models: Organisms/strains**		

Red-bellied piranha (*Pygocentrus nattereri*)	Local dealer or Suma Aqualife Park KOBE	N/A
Red-bellied piranha (*Pygocentrus nattereri*): *gnrh1* knockout	This study	N/A
Head-and-tail-light tetra (*Hemigrammus ocellifer*)	Local dealer	N/A
zebrafish (*Danio rerio*):RIKEN WT (RW)	NBRP zebrafish	ZFIN: ZDB-GENO-070802-4
zebrafish (*Danio rerio*): Tg (*pngnrh1*:RFP)	This study	N/A
zebrafish (*Danio rerio*): Tg (*pngnrh3*:GFP)	This study	N/A
zebrafish (*Danio rerio*): Tg (*pngnrh1*:RFP; *pngnrh3*:GFP)	This study	N/A
Medaka (*Oryzias latipes*)	Local dealer	N/A
Medaka (*Oryzias latipes*): Tg (*pngnrh1*:RFP)	This study	N/A
Medaka (*Oryzias latipes*): Tg (*pngnrh3*:GFP)	This study	N/A
Medaka (*Oryzias* latipes): Tg (*pngnrh1*:RFP; *pngnrh3*:GFP)	This study	N/A
Neon tetra (*Paracheirodon innesi*)	Local dealer	N/A
Glowlight tetra (*Hemigrammus erythrozonus*)	Local dealer	N/A

**Oligonucleotides**		

See Table S1	This study	N/A

**Recombinant DNA**		

pGEM-T vector	Promega	Cat#: A362A
Plasmid: *pngnrh1*:RFP	This study	N/A
Plasmid: *pngnrh3*:GFP	This study	N/A

**Software and algorithms**		

TFBIND	Tsunoda et al.^56^	https://tfbind.hgc.jp/

### Resource availability

#### Lead contact

Further information and requests for resources and reagents should be directed to and will be fulfilled by the lead contact, Shinji Kanda (shinji@aori.u-tokyo.ac.jp).

#### Materials availability

Plasmids and antibodies generated in this study can be requested from the lead contact.

#### Data and code availability

• Sequences of piranha *gnrh1*, *gnrh3* and Head-and-tail-light tetra *gnrh3* data have been deposited at GenBank and are publicly available as of the date of publication. Accession numbers are listed in the key resources table.
• This paper does not report original code.
• All other data reported in this paper is available from the lead contact upon request from the lead contact.

### Experimental model and study participant details

#### Animals

Piranha (red-bellied piranha, *Pygocentrus nattereri*) were obtained from local dealer or Suma Aqualife Park KOBE, and specimen that weighted more than 45 g were used for histological experiments. Male and female gonadosomatic indices (GSI) were 1.27 ± 0.90 and 6.33 ± 2.46, respectively. Head-and-tail-light tetra (*Hemigrammus ocellifer*, weight: 0.93 ± 0.37 g; GSI in male: 1.8, GSI in female: 13.7 ± 3.1) were obtained from a local dealer. RIKEN WT (RW) zebrafish (*Danio rerio*) and medaka (*Oryzias latipes*) were obtained from NBRP zebrafish and a local dealer, respectively, and sexually matured adult with weights > 270 mg (zebrafish) and > 110 mg (medaka) were used. All animals in this study were maintained at 14 h-light/ 10 h-dark at a water temperature of 27 ± 2°C. All experiments were conducted in accordance with the protocols approved by the Animal Care and Use Committee of the University of Tokyo (permission number, 17-1 and P19-3).

#### Artificial fertilization and generation of *gnrh1* KO piranha

Mature female piranha were given intraperitoneal injection of 3 or 5 U/g body weight human chorionic gonadotropin (hCG, ASKA Animal Health, Tokyo, Japan) twice every 24 hours. Unfertilized eggs were obtained by squeezing the abdomen 12 hours after the second injection. After anesthesia with 0.02% ethyl 3-aminobenzoate methanesulfonate (MS-222, Sigma-Aldrich, St. Louis, MO), a portion of the testis was removed through an incision in the abdomen of the male and a testicular suspension was prepared. Fertilized eggs were obtained by quickly mixing the unfertilized eggs and the testicular suspension. To generate KO piranha using the CRISPR/Cas9 system, we designed three types of gRNA at the 5′ side of the sequence of the *gnrh1* mature peptide. A mixture containing 30 ng/μl of each gRNA, 1 μg/μl of Cas9 protein, 100 ng/μl of tracr RNA, 1 ng/μl of EGFP mRNA, and 0.02% phenol red in 1× PBS was injected into piranha fertilized eggs. The designed gRNA regions are shown in Figure S2A. The primers for genotyping are shown in Table S1.

#### Generation of transgenic zebrafish and medaka

The upstream sequences of cloned piranha *gnrh1* or *gnrh3* were connected to zebrafish heat shock promoter as a minimal promoter, dTomato (RFP; for piranha *gnrh1*) or EGFP (GFP; for piranha *gnrh3*) and bovine growth hormone poly(A) signal sequentially and inserted into the pGEM-T vector. For screening in embryos, cardiac myosin light chain 2 promoter of zebrafish, mCherry (RFP; for piranha *gnrh1*) or EGFP (for piranha *gnrh3*) and SV40 poly(A) signal were inserted downstream of reporter construct.^54^ Microinjection of the plasmid into fertilized eggs of zebrafish and medaka was performed. After maturation, they were crossed and F1 embryos were screened based on the fluorescence in cardiac muscle.

### Method details

#### Isolation of coding sequences and genomic sequences

Coding sequences of *gnrh1* and *gnrh3* genes of piranha were identified by 3′RACE with degenerate primers followed by 5′RACE of gene specific primers. Also, partial sequence of *gnrh3* of head-and-tail-light tetra was determined by 3′RACE followed by RT-PCR. The total RNA were isolated from piranha or head-and-tail-light tetra by using ISOGEN (Nippon gene, Tokyo, Japan), and were applied to the SMART RACE kit (Takara Bio USA , Mountain View, CA) according to the manufacturer’s instructions. After cloning into pGEM-T vector (Promega, Madison, WI), sequences were analyzed by a commercial Sanger sequence service. Degenerate primers of well-conserved coding regions that encode GnRH mature peptides. The primers used in these 5′ and 3′RACE are indicated in Table S1. Note that the degenerate primer that amplified piranha (Serrasalmidae) *gnrh1* could not amplify *gnrh1* of neon tetra, head-and-tail-light tetra or glowlight tetra (Characidae; Figure S3C), which is consistent with the fact that a *gnrh1-*like gene is not found in a genome database of a Characidae fish, Mexican tetra. Identified sequences were deposited in GenBank and assigned the accession number LC786488 (piranha *gnrh1*); LC786489 (piranha *gnrh3*); LC785397 (head-and-tail-light tetra *gnrh3*). The isolation of the upstream genome sequences of *gnrh1* and *gnrh3* of piranha was performed using the Universal GenomeWalker™ kit (Takara Bio USA) according to the instructions, and 5.0 kbp (Figure S6) and 3.6 kbp (Figure S7) upstream sequences were obtained, respectively.

#### Preparation of the brain sections

Adult male and female piranha, head-and-tail-light tetra, medaka and zebrafish were deeply anesthetized with 0.02% MS-222 and fixed by perfusion with 4% paraformaldehyde (PFA) in PBS. For piranha and zebrafish, saline was perfused before the perfusion of the 4% PFA. After their brains were dissected out, they were post-fixed by 4% PFA in PBS for more than 4 hours. They were then immersed in 30% sucrose in PBS for more than 4 hours for cryoprotection. They were then embedded in 5% agarose (type IX-A; Sigma-Aldrich) and 20% sucrose in PBS, frozen in *n*-hexane (∼-60°C), and serial frontal sections were prepared at 25 μm thick on a cryostat.

#### Antibodies

Peptides of the precursor sites of piranha GnRH1 and GnRH3 (see Figures S2A and S2B), called GAP sequence, conjugated to KLH were used as antigens, and rabbit polyclonal antibodies against them were produced in this study (outsourced to Sigma-Aldrich). The immunized species and dilution concentrations of the primary antibodies used are as follows; piranha GAP1 antibody (rabbit, 1/10,000) for piranha GnRH1; piranha GAP3 antibody (rabbit, 1/10,000) for piranha and head-and-tail-light tetra GnRH3; anti-RFP antibody 600-401-379S (rabbit, 1/2,000, Rockland Immunochemicals, Inc., Gilbertsville, PA) for RFP; GFP polyclonal antibody A11122 (rabbit, 1/1,000, Thermo Fisher Scientific, Waltham, MA) for GFP. For the secondary antibody, anti-rabbit IgG, biotinylated (goat, 1/200; Vector Laboratories, Burlingame, CA) was used.

#### *In situ* hybridization, immunohistochemistry

For *in situ* hybridization, digoxigenin (DIG)-labeled RNA probes of piranha *gnrh1*, piranha *gnrh3*, head-and-tail-light tetra *gnrh3*, zebrafish *gnrh3*, medaka *gnrh1* and medaka *gnrh3* and fluorescein-labeled RNA probe of piranha *gnrh3* were synthesized with DIG or a fluorescein RNA labeling mix (Roche, Basel, Switzerland). Probe sequences are shown in Figure S2. After 1 μg/ml Proteinase K (Takara Bio, Shiga, Japan) treatment, the sections were hybridized with probes in a hybridization buffer (50% formamide, 3× SSC, 0.12 M phosphate buffer pH 7.4, 1× Denhardt solution, 125 μg/ml tRNA, 0.1 mg/ml calf thymus DNA, and 10% dextran sulfate) at 58°C overnight. After rinsing, for single-labeled *in situ* hybridization (piranha *gnrh1*, piranha *gnrh3*, head-and-tail-light tetra *gnrh3*), alkaline phosphatase-conjugated anti-DIG antibody (1/5,000, Roche) were treated and the alkaline phosphatase activity was detected by 337 μg/ml 4-nitroblue tetrazolium chloride (NBT) and 175 μg/ml 5-bromo-4-chloro-3-indoyl-phosphate (BCIP). For double-labeled *in situ* hybridization (piranha *gnrh1* and piranha *gnrh3*), alkaline phosphatase-conjugated anti-DIG antibody (1/2,000, Roche) and horseradish peroxidase (HRP)-conjugated anti-fluorescein antibody (1/500, Roche) were applied. The activity of alkaline phosphatase was visualized using SIGMAFAST™ Fast Red TR/Naphthol tablet (Sigma-Aldrich). HRP activity was detected by TSA-plus biotin (Perkin Elmer, Waltham, MA), followed by a Vectastain Elite ABC kit (Vector Laboratories), and then streptavidin, Alexa Fluor™ 488 conjugate (Thermo Fisher Scientific).

For immunohistochemistry of piranha GnRH1 and piranha and head-and-tail-light tetra GnRH3, sections were incubated in 0.5× SSC at 95°C for 20 min for antigen retrieval before the primary antibody reaction. Primary antibody was incubated in PBS containing 5% normal goat serum at room temperature for immunohistochemistry of all samples (piranha GnRH1, piranha GnRH3 and head-and-tail-light tetra GnRH3). Afterwards, inactivation of endogenous peroxidase by 0.3% H_2_O_2_ and secondary antibody reaction were performed. The sections were incubated with Vectastain Elite ABC kit and the peroxidase activity was detected by 1 mg/ml 3,3-diaminobenzidine (DAB) and 0.003% H_2_O_2_. For validation of produced piranha GAP1 and GAP3 antibodies, immunohistochemistry was performed using preabsorbed primary antibody with 0.01% KLH and 1 μM GAP1 or GAP3 peptide.

By using transgenic zebrafish and medaka brain sections, double labeling of *in situ* hybridization and immunohistochemistry was performed. Primary antibody against RFP or GFP was treated at 4°C overnight, and for immunohistochemistry of RFP, 1 μg/ml Proteinase K were treated for the antigen retrieval of RFP epitope before primary antibody reaction. After secondary antibody reaction, samples were fixed with 4% PFA, and hybridization with a DIG RNA probe (*gnrh1* or *gnrh3* of zebrafish or medaka) was performed in a hybridization buffer described above at 58°C overnight. After rinsing, peroxidase-conjugated anti-DIG antibody (0.3 U/ml, Roche) were treated, and the peroxidase activity was detected by TSA Plus Cyanine 3 kit (Perkin Elmer) or Alexa Fluor™ 488 Tyramide SuperBoost™ Kit (Thermo Fisher Scientific). Afterwards, the signal of the GFP or RFP was visualized by Vectastain Elite ABC kit followed by streptavidin-conjugated Alexa Fluor™ 488 or Alexa Fluor™ 555 (Thermo Fisher Scientific).

For dual immunohistochemistry, brain sections of transgenic zebrafish and medaka were treated with Proteinase K for antigen treatment (37°C, 10 min). They were then incubated with primary antibody against RFP raised in mouse (1/2,000; RF5R, Thermo Fisher Scientific) and antibody against GFP raised in rabbit (1/1,000; A11122, Thermo Fisher Scientific) at room temperature overnight. After secondary antibody treatment (Alexa Fluor™ 555 conjugated anti-mouse IgG, (1/800; Thermo Fisher Scientific) and anti-rabbit IgG, biotinylated (1/200; Vector Laboratories) for 2 hours, the sections were incubated with Vectastain Elite ABC kit followed by streptavidin, Alexa Fluor™ 488 conjugate. For nuclear counterstaining, 0.00025% methyl green solution diluted in PBS was treated for 10 min.^55^ Fluorescence-labeled sections were mounted on coverslips with CC/Mount (Sigma-Aldrich). Sections with DAB labeling were captured by an upright microscope, Olympus BX-53 equipped with NOA630B or NOA2000 (Wraymer, Osaka, Japan). All the fluorescence-labeled sections were observed under a confocal microscope, Olympus FV1000. Contrasts and brightness were modified with ImageJ (NIH, Bethesda, MD).

#### Transcription factor binding sites analysis

We determined potential transcription factor binding sites for upstream sequence of piranha *gnrh1* and *gnrh3* by using TFBIND website (https://tfbind.hgc.jp/).^56^ The cut-off value was set to 0.9.

### Quantification and statistical analysis

There is no statistical analysis in this paper.

## Supplemental information

Document S1. Figures S1–S8 and Table S1

Table S2. Transcription factor prediction by TFBIND for upstream sequences of piranha *gnrh1* and *gnrh3*, related to Figures 3 and 4
