# Supplementary material for: Long-lasting redundant gnrh1/3 expression in GnRH neurons enabled apparent switching of paralog usage during evolution
Source: iScience. 2024 Feb 22;27(3):109304. doi: 10.1016/j.isci.2024.109304 (PMC10924128; doi:10.1016/j.isci.2024.109304)
Supplement: Document S1. Figures S1–S8 and Table S1 [file mmc1.pdf]

**iScience, Volume *27***

## Supplemental information

# Long-lasting redundant *gnrh1/3* expression in GnRH neurons enabled apparent switching of paralog usage during evolution

**Chika Fujimori, Kohei Sugimoto, Mio Ishida, Christopher Yang, Daichi Kayo, Soma Tomihara, Kaori Sano, Yasuhisa Akazome, Yoshitaka Oka, and Shinji Kanda**

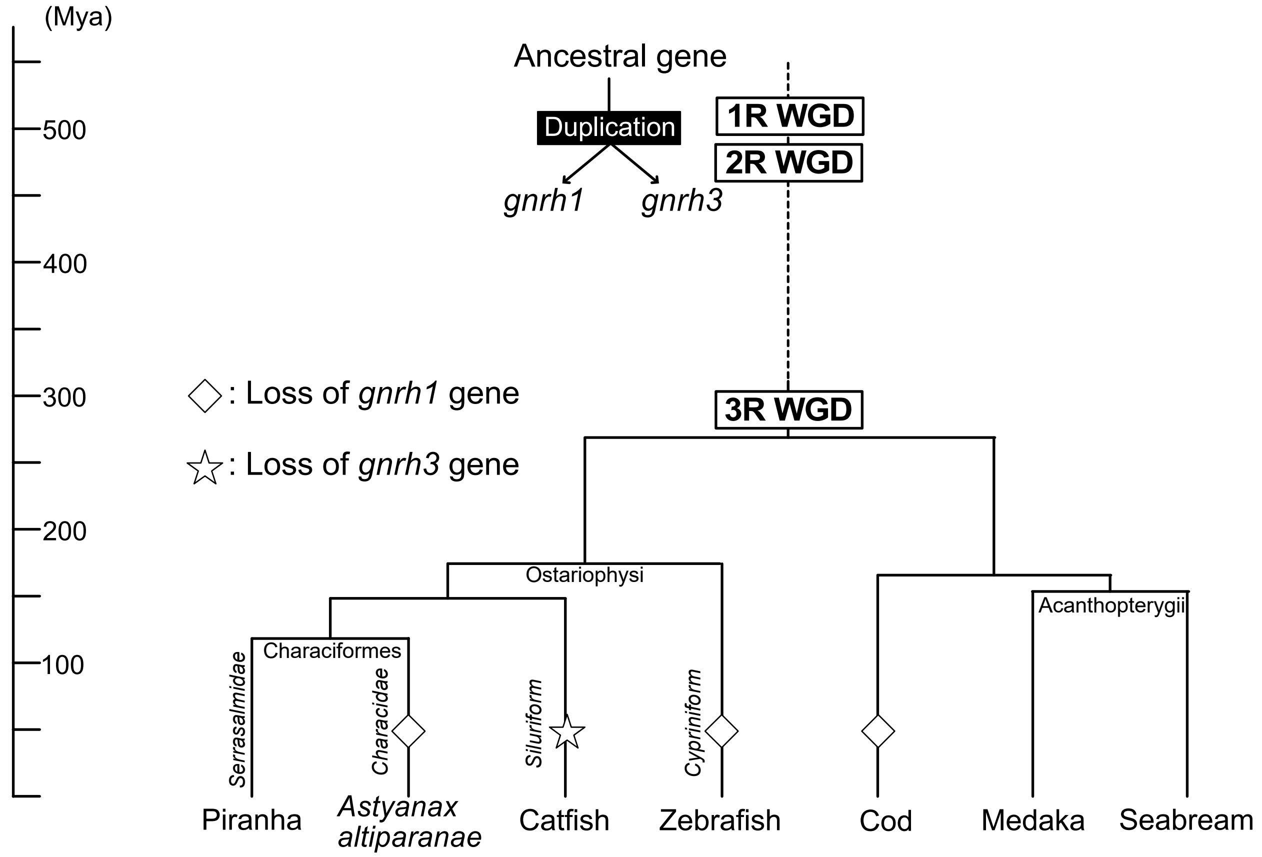


| | Piranha | *Astyanax altiparanae* | Catfish | Zebrafish | Cod | Medaka | Seabream |
|---|---|---|---|---|---|---|---|
| *gnrh* paralog | *gnrh1* *gnrh3* | *gnrh3* | *gnrh1* | *gnrh3* | *gnrh3* | *gnrh1* *gnrh3* | *gnrh1* *gnrh3* |
| (Reference) | (This study) | [S1] | [S2] | [S4] | [S6] | [S7] | [S9] |
| Hypophysiotropic *gnrh* | ? | *gnrh3?* | *gnrh1* | *gnrh3* | *gnrh3?* | *gnrh1* | *gnrh1* |
| (Reference) | | | [S3] | [S5] | | [S8] | [S10] |

**Figure S1. Phylogenetic tree of teleost species and their hypophysiotropic GnRH paralogs, related to introduction and Figure 5.**

The *gnrh1* and *gnrh3* genes were arisen from an ancestral gene during the first/second-round (1R/2R) whole genome duplication (WGD), which occurred ~550 million years ago (Mya). Many teleost species have hypophysiotropic GnRH1 neurons as the main regulator of gonadotropin release in Acanthopterygii. However, in Cypriniform and Characidae species as well as Atlantic cod, *gnrh1* has been lost and *gnrh3* is expressed in hypophysiotropic GnRH neurons instead. Since three forms of GnRH peptide are expressed in a Serrasalmidae species, pacu, we expected species in this group, including piranha, may have both *gnrh1* and *gnrh3*.

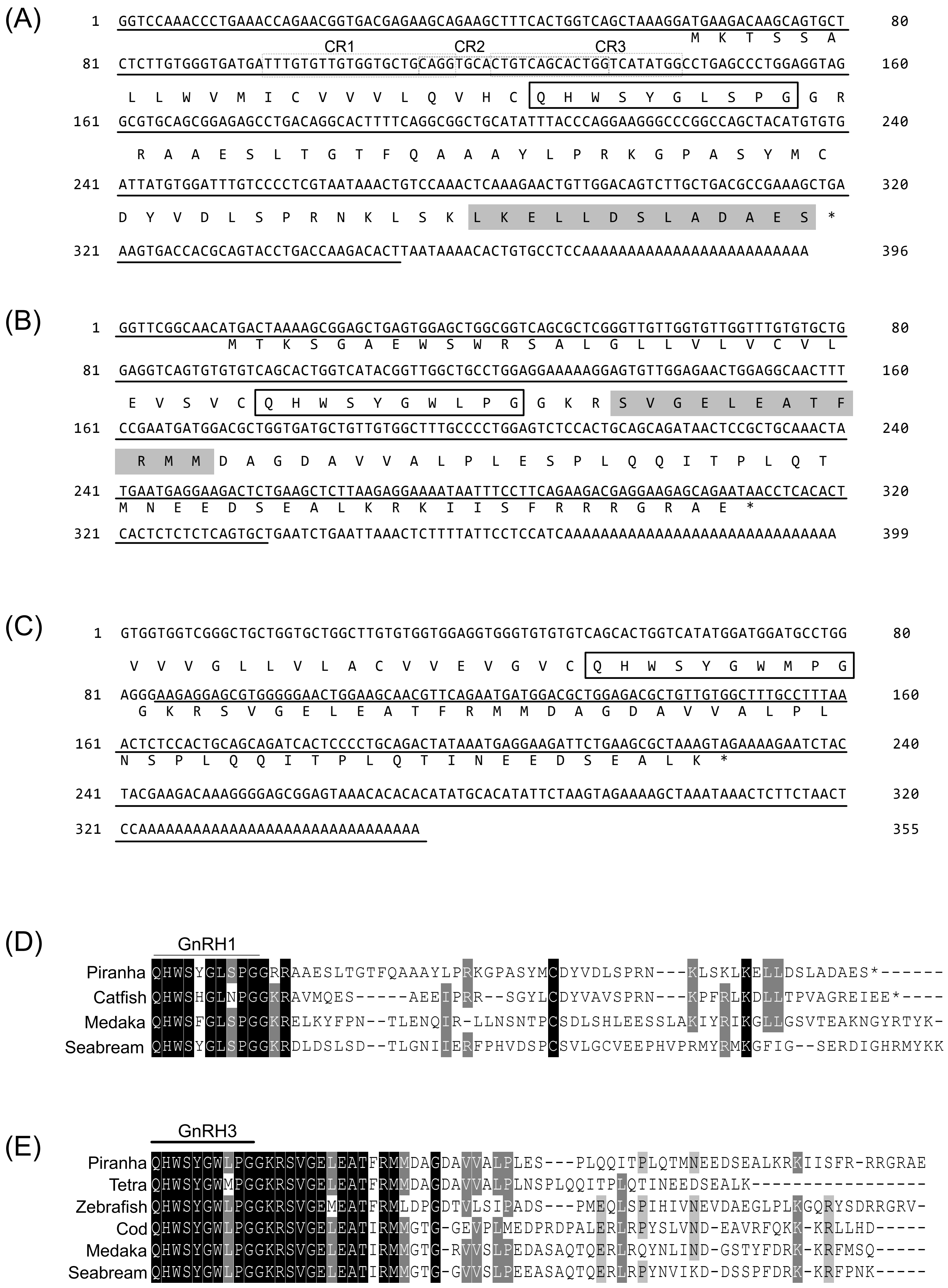
(A)
1 GGTCCAAACCCTGAAACCAGAACGGTGACGAGAAGCAGAAGCTTTCACTGGTCAGCTAAAGGATGAAGACAAGCAGTGCT 80
M K T S S A
CR1 CR2 CR3
81 CTCTTGTGGGTGATGATTTGTGTTGTGGTGCTGCAGGTGCACTGTCAGCACTGGTCATATGGCCTGAGCCCTGGAGGTAG 160
L L W V M I C V V V L Q V H C Q H W S Y G L S P G G R
161 GCGTGCAGCGGAGAGCCTGACAGGCACTTTTCAGGCGGCTGCATATTTACCCAGGAAGGGCCCGGCCAGCTACATGTGTG 240
R A A E S L T G T F Q A A A Y L P R K G P A S Y M C
241 ATTATGTGGATTTGTCCCCTCGTAATAAACTGTCCAAACTCAAAGAACTGTTGGACAGTCTTGCTGACGCCGAAAGCTGA 320
D Y V D L S P R N K L S K L K E L L D S L A D A E S *
321 AAGTGACCACGCAGTACCTGACCAAGACACTTAATAAAACACTGTGCCTCCAAAAAAAAAAAAAAAAAAAAAAAAAA 396
(B)
1 GGTTCGGCAACATGACTAAAAGCGGAGCTGAGTGGAGCTGGCGGTCAGCGCTCGGGTTGTTGGTGTTGGTTTGTGTGCTG 80
M T K S G A E W S W R S A L G L L V L V C V L
81 GAGGTCAGTGTGTGTCAGCACTGGTCATACGGTTGGCTGCCTGGAGGAAAAAGGAGTGTTGGAGAACTGGAGGCAACTTT 160
E V S V C Q H W S Y G W L P G G K R S V G E L E A T F
161 CCGAATGATGGACGCTGGTGATGCTGTTGTGGCTTTGCCCCTGGAGTCTCCACTGCAGCAGATAACTCCGCTGCAAACTA 240
R M M D A G D A V V A L P L E S P L Q Q I T P L Q T
241 TGAATGAGGAAGACTCTGAAGCTCTTAAGAGGAAAATAATTTCCTTCAGAAGACGAGGAAGAGCAGAATAACCTCACACT 320
M N E E D S E A L K R K I I S F R R R G R A E *
321 CACTCTCTCTCAGTGCTGAATCTGAATTAAACTCTTTTATTCCTCCATCAAAAAAAAAAAAAAAAAAAAAAAAAAAAAAA 399
(C)
1 GTGGTGGTCGGGCTGCTGGTGCTGGCTTGTGTGGTGGAGGTGGGTGTGTGTCAGCACTGGTCATATGGATGGATGCCTGG 80
V V V G L L V L A C V V E V G V C Q H W S Y G W M P G
81 AGGGAAGAGGAGCGTGGGGGAACTGGAAGCAACGTTCAGAATGATGGACGCTGGAGACGCTGTTGTGGCTTTGCCTTTAA 160
G K R S V G E L E A T F R M M D A G D A V V A L P L
161 ACTCTCCACTGCAGCAGATCACTCCCCTGCAGACTATAAATGAGGAAGATTCTGAAGCGCTAAAGTAGAAAAGAATCTAC 240
N S P L Q Q I T P L Q T I N E E D S E A L K *
241 TACGAAGACAAAGGGGAGCGGAGTAAACACACACATATGCACATATTCTAAGTAGAAAAGCTAAATAAACTCTTCTAACT 320
321 CCAAAAAAAAAAAAAAAAAAAAAAAAAAAAAAAAAA 355
(D)
GnRH1
Piranha QHWSYGLSPGGRRAAESLTGTFQAAAYLPRKGPASYMCDYVDLSPRN---KLSKLKELLDSLADAES*------
Catfish QHWSHGLNPGGKRAVMQES-----AEEIPRR--SGYLCDYVAVSPRN---KPFRLKDLLTPVAGREIEE*----
Medaka QHWSFGLSPGGKRELKYFPN--TLENQIR-LLNSNTPCSDLSHLEESSLAKIYRIKGLLGSVTEAKNGYRTYK-
Seabream QHWSYGLSPGGKRDLDSLSD--TLGNIIERFPHVDSPCSVLGCVEEPHVPRMYRMKGFIG--SERDIGHRMYKK
(E)
GnRH3
Piranha QHWSYGWLPGGKRSVGELEATFRMMDAGDAVVALPLES---PLQQITPLQTMNEEDSEALKRKIISFR-RRGRAE
Tetra QHWSYGWMPGGKRSVGELEATFRMMDAGDAVVALPLNSPLQQITPLQTINEEDSEALK-----------------
Zebrafish QHWSYGWLPGGKRSVGEMEATFRMLDPGDTVLSIPADS---PMEQLSPIHIVNEVDAEGLPLKGQRYSDRRGRV-
Cod QHWSYGWLPGGKRSVGELEATIRMMGTG-GEVPLMEDPRDPALERLRPYSLVND-EAVRFQKK-KRLLHD-----
Medaka QHWSYGWLPGGKRSVGELEATIRMMGTG-RVVSLPEDASAQTQERLRQYNLIND-GSTYFDRK-KRFMSQ-----
Seabream QHWSYGWLPGGKRSVGELEATIRMMGTG-GVVSLPEEASAQTQERLRPYNVIKD-DSSPFDRK-KRFPNK-----

**Figure S2. Alignment of piranha *gnrh1*, *gnrh3* and head-and-tail-light tetra *gnrh3*, related to Figure 1 and Figure 2.**

(**A-C**) Nucleotide sequence and deduced amino acid sequence of piranha *gnrh1* (**A**), *gnrh3* (**B**) and head-and-tail-light tetra *gnrh3* gene (**C**). Each box shows mature GnRH peptides. Three dotted boxes (CR1, CR2, CR3) in (**A**) represent gRNA sequences used for generation of *gnrh1* knockout (KO) piranha. Antigen sequence for antibody production is shaded in gray. Underlines show probe sequences for *in situ* hybridization. (**D**) Alignment of the deduced amino acid sequences of mature GnRH1 and GnRH-associated peptide (GAP1) in piranha with those in channel catfish (catfish), medaka and gilthead seabream (seabream). Mature GnRH1 sequence of piranha is identical to that of gilthead seabream. Conserved amino acids in all 4 species and 3 species are shaded black and gray, respectively. (**E**) Alignment of the deduced amino acid sequences of mature GnRH3 and GnRH-associated peptide (GAP3) in piranha and head-and-tail-light tetra with those in zebrafish, cod, medaka and gilthead seabream (seabream). Mature GnRH3 sequence of piranha is identical to those of all species aligned except for head-and-tail-light tetra (Tetra). Conserved amino acids in all 6 species and 4 species are shaded black and gray, respectively.

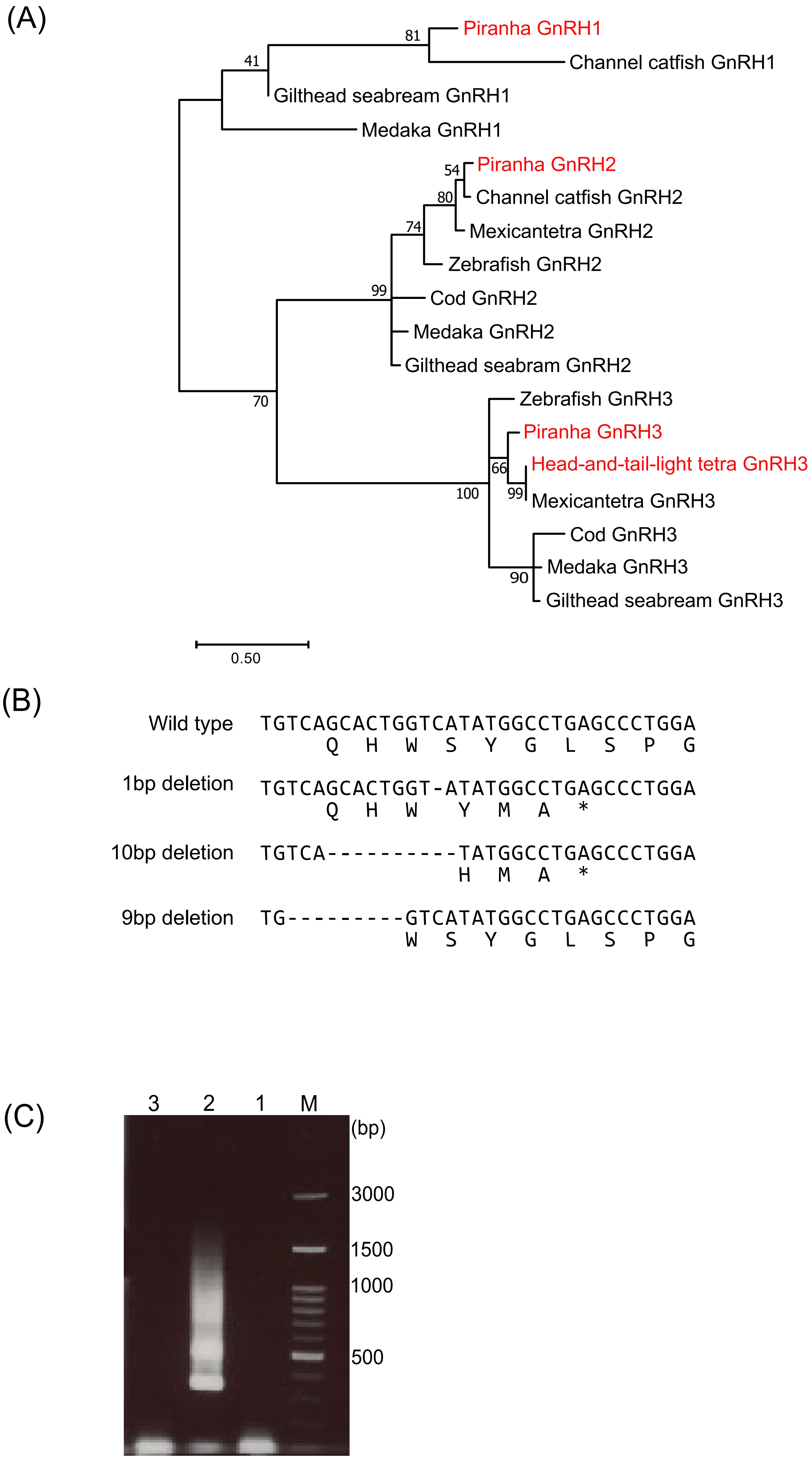

(A)
Piranha GnRH1
Channel catfish GnRH1
Gilthead seabream GnRH1
Medaka GnRH1
Piranha GnRH2
Channel catfish GnRH2
Mexicantetra GnRH2
Zebrafish GnRH2
Cod GnRH2
Medaka GnRH2
Gilthead seabram GnRH2
Zebrafish GnRH3
Piranha GnRH3
Head-and-tail-light tetra GnRH3
Mexicantetra GnRH3
Cod GnRH3
Medaka GnRH3
Gilthead seabream GnRH3
81
41
54
80
74
99
70
66
100
99
90
0.50
(B)
Wild type TGTCAGCACTGGTCATATGGCCTGAGCCCTGGA
Q H W S Y G L S P G
1bp deletion TGTCAGCACTGGT-ATATGGCCTGAGCCCTGGA
Q H W Y M A *
10bp deletion TGTCA----------TATGGCCTGAGCCCTGGA
H M A *
9bp deletion TG---------GTCATATGGCCTGAGCCCTGGA
W S Y G L S P G
(C)
3 2 1 M
(bp)
3000
1500
1000
500

**Figure S3. Phylogenetic tree of *gnrh1* and *gnrh3*, information of the knockout, and a PCR result, related to Figure 1 and Figure 2.**

(**A**) Phylogenetic tree of GnRH paralogs in piranha, head-and-tail-light tetra, and other teleosts (Maximum likelihood). The number at each node shows the percentage of the bootstrap value for 1000 replicates. Scale bar represents 0.50 substitutions per site. (**B**) Genomic and deduced mature peptide sequences of wild type and KO *gnrh1* gene. Both 1-bp and 10-bp deletion mutation resulted in a frameshift and a premature stop codon (asterisk). In all mutations, it is expected that functional GnRH peptides are not produced. **(C)** Detection of *gnrh1* mRNA was failed in various Characidae fish cDNA. Degenerate primers that amplified piranha *gnrh1* failed to amplify *gnrh1* of neontetra (lane1), head-and-tail-light tetra (lane2) and glowlight tetra (lane3). Note that the bands observed in lane 2 are nonspecific amplificons. M, DNA markers (bp).

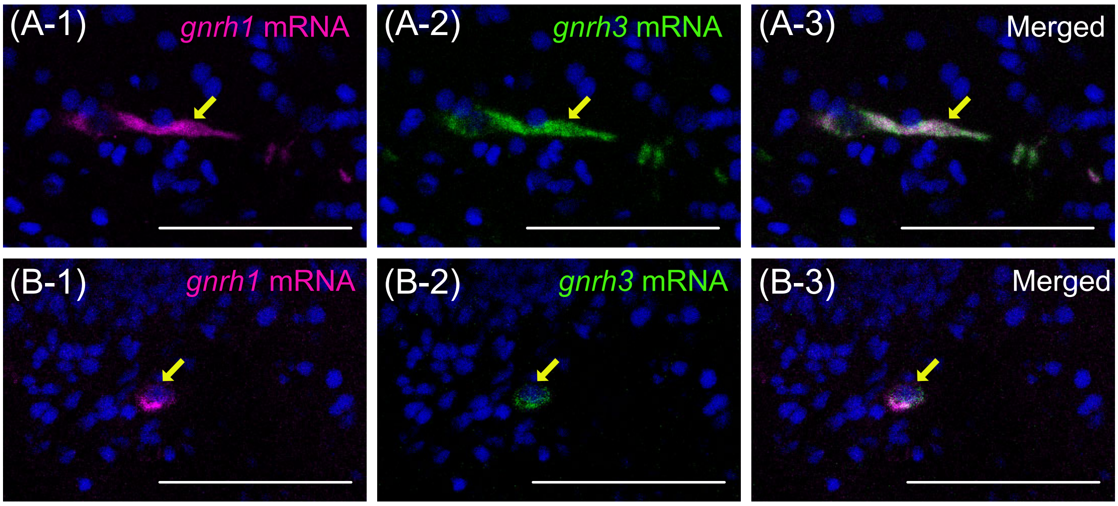


**Figure S4. Other representative sections of double-labeled *in situ* hybridization of *gnrh1* and *gnrh3* in piranha POA, related to Figure 1.**

(A, B) Double *in situ* hybridization indicates that co-expression of *gnrh1* and *gnrh3* is observed in many *gnrh*-expressing cells in POA. Magenta signals in (A-1), (A-3), (B-1) and (B-3) show *gnrh1* mRNA, and green signals in (A-2), (A-3), (B-2) and (B-3) indicate *gnrh3* mRNA. Nuclear counterstaining using methyl green is shown as blue signals.

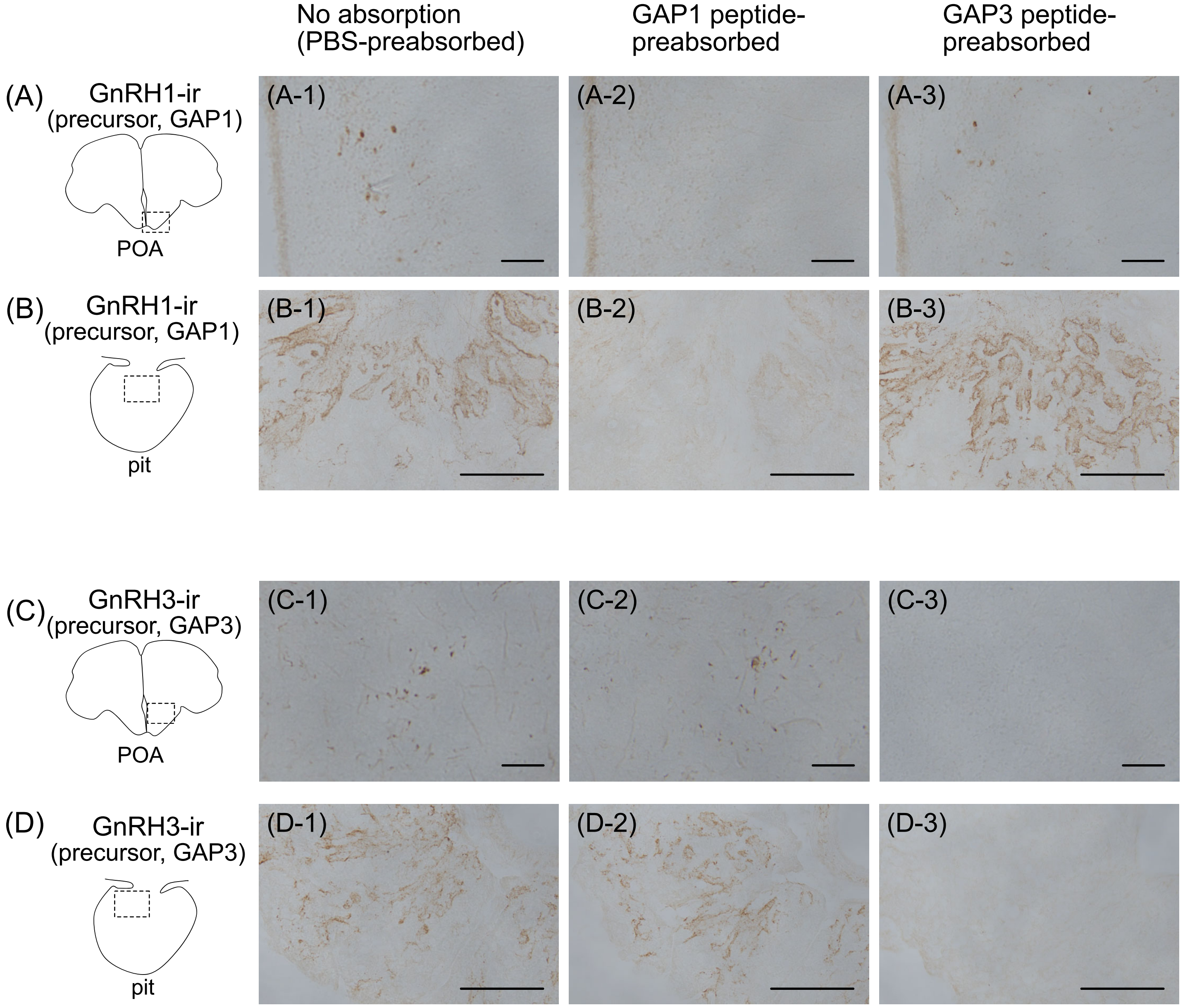


**Figure S5. Specificities of produced GnRH precursor antibodies, related to Figure 1 and Figure 2.**

(**A, B**) Immunohistochemistry for GnRH1 precursor of transverse section of piranha POA (**A**) and pituitary (**B**). Immunoreactive cells and fibers are labeled using a GnRH1 precursor antibody (**A-1, B-1**) and are not labeled with a GnRH1 precursor antibody preabsorbed with GnRH1 precursor peptides (**A-2, B-2**). (**A-3, B-3**) In immunohistochemistry using a GnRH1 precursor antibody preabsorbed with GnRH3 precursor peptide, immunoreactive cells and fibers are labeled. (**C, D**) Immunohistochemistry for GnRH3 precursors shows that immunoreactive cells and fibers are labeled in the POA (**C**) and the pituitary (**D**) when preabsorbed with PBS (**C-1, D-1**) or GnRH1 precursor peptide (**C-2, D-2**), but are not labeled when preabsorbed with GnRH3 precursor peptide (**C-3, D-3**). Scale bars, 100 μm.

```
1       AATTCTTTCTAGTCATCGTAGTTTTAAGACTGTAAAGTTTGGTCAGTAGGAGGTGGCCAAATCTGCTCTGTTTTTTTCTC    80
81      ACGGATTTGAATAAGCGTCAGTGGGTTTTTTGCCATTGATACTGCTACACAAAACACAAATCTGTGTCACATATGAAGAA    160
161     AAAGATTGGACATGGGCCACTTTTACCTGCAGTGTGAATGTAACTTATGAATCTTTAAAAAGCTCTCTTGCGTAGTAACA    240
241     GTAGCCTTGCTGTACCAGTACATATAGATCGGAGCATTGAGTTTGTGGTTTGCCAATGACTGGCAGTTAATGCATTCGTG    320
321     TTTCAGAGTTCTGGAATATTTTAGGAGTTTAAGACTCTGAAATTGGTGAAATGTTGAAGTTAGGCAGAGCAGCCTTTTCT    400
401     GAGGCTTTTCTTGATTTTCAAGGGCACGTCAGGTTCAGTTTGAAGCCATATTATATCGTGCATGCTTAAGGAGGCAAAAT    480
481     CTTGATATCTGTGTAGCATATGAAAGAAAAGGGTGATGGATTCCATAACCTCATATTTCTATATCAATTTTCTAGGTTAG    560
561     GCATGCAGTTAAATATGCAATATAAATGTATTAGTGCTGTCTGTTTTTGACGTTTCTGTTGTGCAAATGCAAATGACAGC    640
641     AGTTTGTATCACTTTCGTTTGTTTGCTGGTTTGTTCCAAATCTACCTCTCACTTAGGATACTGACCGTTTTTTTTTTTTT    720
721     TTTTTTTTTTTTTTTTTTGCTGTTTTTGTGAGTTTGGGTTTTTTTTGGCCATTGTCAATGCTGTTCTCTTGGATTCTCAT    800
801     GCGAAGGCTTGTGCTGACTGTGAGTGGATAGACGCTAGGATGTTAAATGAAAGATAAATTACCAAATTGCTGTTGTATTT    880
881     TTGAGTTATGCACACCCTGTGGCTATCAACTCGAGGTATAATGATACTAGCCTATTAATTAATATGCAGAACAGTTCACC    960
961     AGTAGCAAGACAACACTGCTTTGAATGATGATTTGTGTATTGTGTGACTGTTTCTCAACAGATATTTCTTAGTTTTTTTT    1040
1041    CTTGACATTATTAGTAGTGTAACTACTAATAAAAATGTCTTTGTTCAGGCAAAACCAAAAAGCTTTGAATGGGGTGCTTA    1120
1121    CCTGTCAGATAGAGTGCTTCCCTCAGCGTAATCGCATAAAACCGTTCCTTGCTGATTTGACAGGCCTGAAATCATTGTCA    1200
1201    CTGACCGCATTAGCATCAAGTCGTGGTCATCCGTATGCCCACTTAGCACTTATTTATATTGTCCACGCTTTGTCTGTAAG    1280
1281    CCTATTACAATAATATGTTTGTGTAGTGTTTGCTGATTCCTTACCTACTGCCCTGCAGTTCATGTAATGCTTTCAATGTT    1360
1361    AATATTATTCCCATGGGGTCAGACCTGCTCTTGATATAAGAATGCATGTCTGGAGACACTTGTTAAGCTTTTGCACACCC    1440
1441    AATCACCTGCAGACAAGGGTACGCTCCCTCTAAAACCCACCCAGATGTCTCTTGTCCAGGTCTAGAACACGAGCAAAACA    1520
1521    AGTTAGCTAGCAAAACGCTATCGTTAGCTAATTAAACTTGTTAACGCTTGCCGGGGGCTCATCTTCTTTTTTACTGTGTG    1600
1601    TTCTCCACACTTGCTGCTCACATCTACATGCTTATTTGGAGTTAATATGGGGGACTTTTATGTTACATCCATGTGCTAAC    1680
1681    AACAGGCTTCTTATACTTTGGACAGCAAACGCACAAAATTAGCAAGTCACTTTTTTTATTTTTTATTTTTTTGAGCTTGT    1760
1761    TTCCACCCAAATATATGCATGTTCCCAAAATTTCTTGTAGTTGTCCCCTAATGTTCTCGTTACGAGATCATGTCTGGCCC    1840
1841    CCATGCGAGTGTTAATATCAGCTGGATTATTGCTGTGTGGTTAAAAACTAAGTCAGACCTGTTTTCTGGCAATAGCTTAA    1920
1921    AGTTAATGGGATTCAATATGCTGTTACTTCTCTGATGTATGTAATTGTAGGTACTCCTGTTGAATGAAGGCAATCAAAGC    2000
2001    AGACTTCTGCACTTTAAGTGTGAGTCTAAATTTACTGGACCCTTGACGAAAGGGCCCAGGCTATTTATGTATGTATGTTA    2080
2081    TGTATGTAATCAGGCTATATTTATGGCAGGTGAAAAAAGAGATGTCCTCTACTTTCACCTCACTATCTTGCACTCACTAT    2160
2161    TACGGAAGTGTATTTCCATAAACAGACTGTTATAATCAAACTCGTCTTGTGTCTTCATTTTTCCCGTTTGTGTTTTTGGT    2240
2241    CTTTAATCTTGTTGTTTGAGTGAACACTCCAACTCTGTACGAGTGACGCTCAGAACGAATCATTCAAACTGCTCCATTAA    2320
2321    AATGCTTCAGTTATTCTAATCAGGTATTTTGCACGTGATAAACGCTGCAAGCGTTACAAAGAAAGGGGAACTTCAGTCGG    2400
2401    AATTTTGCCCCTGACTGGTTAGAAAGTGTCTTTGACTGGCCCAAGAGCTGTTTATGATCCCAAATTCCCAACTTAAAGGG    2480
2481    GAATAATTGAGCCTTTGAGATGTAAGCAAAGTCATTCTGAGCAGTTTGATGCGAAACGGTTCATTGTAGAGAAACTGCCT    2560
2561    CAGATTTCTTTATGGTGGTGATTGAAACCAGGAGTCACAGTGTCTACAGCATAAATCCACTTTTTTACTATCCAAAATGA    2640
2641    ACTGTACACAAGTTTTCAGACATTTTATGCCTAATTTGCATAATCACAAATTTTAAAAAGGCATCAGCCGTGTCTTCAGA    2720
2721    ACCACTGTGAGCAAGCTTTAAGATTTGATACATTTTTCTGGATTGAATCATTTCACACCAAACCACTCTGAAGGACTATT    2800
2801    TACAGCTTAACCATTTCATTATGTAGAAAAATAGACAAATCAGTGGAAGTCCAATCCAAAAAAAGGTTCAGCGTGGCTGG    2880
2881    GGACTTTCATTCATTCAACAAAGCAGGAGCTTGTGCTCAATTGTTTGGAGACTGAGCTCCACTGATTAAAGGTGCCGTGT    2960
2961    GTAAGATGTAGTGACATCTGGTGGTGAGGTTGCAGATTGCAACCAGCTAAATATTACTCTTAAATATCAATGTAACTTTT    3040
3041    GTGTCTTCATGTTTTCGCTTCTTGGATGATGATCCACCCTCCTTTGCTTTTTCTTGTGCCGCAAAATACGATCCTCCATT    3120
3121    TAATAAAGTTTGGGTCCTCAAACTTCTCTCAACACAATGATTAGCCAGCACTGGCAGCAGCCACTGATTCTTCTCTCCAC    3200
3201    GTCTGTACCACTAAATCAGCAACATAAACACGGCAGACGCTCTGGCCTACTGTAGAAACATGGCAGCGCAGCATGGCTGT    3280
3281    TGGAAGAGGACCCACTCCCTGTAGATTTTAAGGGCTCATTCTAAGTGAACGAAAACACAACAAGCTATAGTTACAGGTGA    3360
3361    TAACGCACTAATGAAAACAGGGTTTTGTATACTATATTTTGCTTCTGCCAATAGACTTCTTACACACTGCACCTTTAAAC    3440
3441    GCTCCAGCTTATTTGGAATGAAAATTTGTAGCCACACCAGCTCTGCGGATAAAATTGTACACTCCTGTTTTAGATGGTGA    3520
3521    AACAAACTATAGACCAAAGTGCACAGAAAGTAAGACATATTTACTTACAATGTATCCATCCATCCATCCATCCATTTTCT    3600
3601    AAGCCGCATCTCCGTCAGGGTCGCGGGGGGAAGCTGGAGCCTATCCCAGCAGTCTTCGGGCGGAAGGCAGGATACACCCT    3680
3681    GGACAGGTCGCCAGTCCATCACAGGGCATACACACAGACACAGACAGTCACTCACACCTAGGGACAATTTAGCACACCCA    3760
3761    ATTGGCCTGACTGCATGTCTTTGGACTGTGGGAGGAAAGAACCCGGAGGAAACCCACGCAGACACGGGGAGAACATGCAA    3840
3841    ACTCCACACAGAGAGGACCCCGGTCACCCGGCCGGGGAATCGAACCCAGGCCCTCCTTGCTGTGAGGCGACAGCGCCACC    3920
3921    GTGCCGCCCTTACTTACAATGTTTTTTTTTTTTTTCTACTTACAATGTAGAAATAAAAAAACCAACATAGGCCATAGAATT    4000
4001    GTTTTGAACTCACGTCCTGTTGTCTTGAAATCAACCAGAATAAAGCAAAAGAAATTTCAAACTCTTTGCAAAGACTTAGC    4080
4081    AGAAAGTATAGTGTTATATTAGAAAGCCTGGCACAACTGCACTCCTGGTCAATCACTTGGAGTTCAGTCAGTACCGTTGA    4160
4161    CTCTTCTCATTCACACTTGGGAGCACTTTAAAAACTTCAGCATTAGCCTTCTCTGAGTAATCTCTTAAAAAGGCTCAGTG    4240
4241    GGAAGTGTCTGAAAGGTGCTTACTTCCGTCATCCTTTCATTCTGCTCTTAGCCCCCTGCAGTGAGGGGCTGGAGAAGCGA    4320
4321    TTGCGTGAGACGAGCAAGAGCTCTATAAGAACACTAAAGCTGAGAGCAGGAGTGTCCAAACCCTGAAACCAGAACGGTGA    4400
4401    CGAGAAGCAGAAGCTTTCACTGGTCAGCTAAAGGTAAGTCAGATCCAAGTACGTGTCCTTCTGTTGGCACACTTAGAGCA    4480
4481    CTGGTTCACAAACCAGTCCTCAGGGCCTCTGGATGTTCCAGGTTACTGATCAGAATACCTGATGAAAGGTTTGAAAACTA    4560
4561    ATGCCTTTGAGCAATATTTAGCAGATCAGTGTCCCACAGTTCAAAGAGCTTAGACTGCTTTTGCTGGACAGTTCTGATCC    4640
4641    TAAATTTTAAAGACTTTTATTTTAAAAGGGGGTATAGGTTTAATTTGAAAAGCTAACATCCAAATTTATCTTGGAAAGCC    4720
4721    ACAGTTTCCCTTGATATCCCCTTTGGTATCAAGGCTTTTATGTTGAAAAGCCTTATTTATCCCCAATACCTGAGTATTGA    4800
4801    GACTTTGGAAAGCTACAGTTTCTATGATATTTGGGCATCTTATATTTTTAAAAAAATGCATATTTAACTCAATTGAGTGG    4880
4881    TTTTCAATGTCTGAAGGTGGCTGTTTTCTGTTGCTCTTTTCAGGATGAAGACAAGCAGTGCTCTCTTGTGGGTGATGATT    4960
4961    TGTGTTGTGGT
```

**Figure S6. Upstream sequences of piranha *gnrh1,* related to Figure 3 and Figure 4.**

The upstream sequence of piranha *gnrh1,* which was used for generation of transgenic medaka and zebrafish, Tg(*pngnrh1*:RFP).

1 TTTAAAACAATCATTCCTACGCACAGTGAACTAAGTGCTGTTTCTTAGAGATGAAGTGTGTCTCAGTTTAACTGTTTAGT 80
81 TATCTTGTTTGTTGTTTTCAGTGAACTGACCGCTGAGTGGAGCGCATTCAGACAAATTCAGTTTAACCGATTCAGTTTTT 160
161 CAGTATTTAATTTGTCAAACGACGTCACATTAATTTTATAACACTTAATTTTTTTTATTTGAGTTATTTGTTTACCTGTG 240
241 CACCAGTCATTAAACTCTTGGAAAAATAAATCAAAATCATTTCTTCCTGGTCCTGAGTCTTTCAACCACCACTCAGCTAT 320
321 CCTGCAACACATTATTGTACAGTAAGAAGAAATCACAGAAATTACACATTTCATTTTAGATCTTTATTTGGTAATACAAT 400
401 GTGCATATGTGTGTTTGTGTGTGTGTGTGTGTGTGTGTGTGTGTGTGTGTATGTGTGTGTGTGTGTGTGTGTGTGTGTGT 480
481 GTGTGTGTGTGCGCATGCATGTTGAGTCCGAAAGAGGCAAAGCGGTCCACCTTCACATACTGTTTACATAGTATAATAGT 560
561 ATACATTTAAAATAACCCTAGGATATTGCTATATTGCTTAAAAACTGTACTTACATCAGAGAGTGAGGAAACCATGCTAC 640
641 TCTGTGATGCTCCGTGCAGAGGGTCTTTGACCTAGTAGACAACCCATCTCCATATGAATGGACTGTAAAAAGGAAAAAAG 720
721 TAAATAAATCCCACATTCTTGTTGATACGGCGCCATGAGGTATAGAGATGCTTGAGGTAAATTTGAAAATTAATTTCATT 800
801 GAGCTGTATCCTAAGAAGCATGACAGAAGGATTATGTTCAATAAGAATAAAAGATGAGCGCAGACACATAAAGAAGTACA 880
881 AAGATGACGCAGCACTCTCCTGAAATGTGCTACCACTCATTCCAACATTCAAAACAGAACAGCACCCCCTGCTGTGAGTA 960
961 TCAATGCAAACCCAAATCCTCATTTCATTCACAAAAGTAGTTCACCTCAAATGTCACAATAGTCAGTTACTCAAAATCTG 1040
1041 CACAAGAAGATTGGCAGGAATTTGTGAATTCAGGCTAAGCGTGAAGTTACTTTTCATGCACTGTTAGTGATAAAGTTTCT 1120
1121 GTGCAGGTATATTTTCCCTCAAAGGTACAACAGTGGTTTTAAGGTCCAATTGTGAACCTTAAATAAGTTTTTTCCGGCTG 1200
1201 AAAAATACATATTTTCACGTTTTCATAAGCTAGTGCTTTAAAACAGAACAATAAAATAAAAGCCTGGCGATGAGACGGCG 1280
1281 TGTGCAGAGTCAATACAAATATCATTTCAGTGTATTATAGTACAGTTATGAACCCTGACTAAAGGCACAGAGATGTACCC 1360
1361 TTGAGGGTTCCATCCTAGTGACAAGAGAATAGTGCCCCAGTGGCAGTTTAGTACCTTTATTTATTAAGCGTACAATCACA 1440
1441 AATAAGAAAAGAAAAATTCTCCAAAAAGTTTAGAAACTGGTCCTTCAACATTCATAAAACATAATAAAGAAGAGCATCTC 1520
1521 TAAACATCTCACTGCCAGTAACAAAGTAGAGATGGACATAATCCCCCATCACAGCACAGAAGAAACATTCATTAATGTTG 1600
1601 AAACACATTTTCACATGAATTTACATATTTCTTGTCTGGTCATGATCTGCTACATTAAACAGGACACTATAACAGACAAG 1680
1681 ACTGCGTGGTCAGATTAACCCCTACTTAAATAATTTAGGTCCTGAAATTACAAAAGTTTTACCAAAATGAACTGATCATA 1760
1761 ATAAAAGACTACTATCACTATATCTATGGGTCACATCTCCTTGCCCTTTGGGCCTAGAGCCATCAAATTCAGATATGAGA 1840
1841 AGCATGCCGATGGGACGACAAAATGTTGGGAGTTTTGTAGCTCAGATACCAAGCTACTCACTAGGGACCTCAGTACAAAT 1920
1921 GGGCTGAGTTTTTCTTAGGTTATAGAAGTGTGTAATAAAACTTTGGACCTGTCAGTGGGCCCTGATTATTTAACGCATTA 2000
2001 AGTGCACCAATCATGTGTGAGATTGGGGGCCGAAAGGTGGGTGGGAGTATTCACATATTAATAGGCCCTTTTCACCTTCC 2080
2081 CACATTTTTTCTGCTGGGCGTGAGCTTGAAGCCTCATGTCATTTTCGGTGGTTTGTCCTCTGATGACAAGCAGAGGCGAA 2160
2161 ACAGGTTTAACAAAGGAATAAACTTAAAAGAACCTCTCTGTCAACATTTCACCACTGCTGACTAATGTTAATGTGCAGTT 2240
2241 TATGCTTTAAAGTTTTAAAGAGTTTTCATTTTCTCCATCAATAACTATCAGTATCTGAGAGAGTAAATGTTCACAGGGAA 2320
2321 CAGAGGTTCGTTCACTGGAGTCTGTGCCTGTGTAATATGCCTACAATATCATTACGGACCATCATAACCCAGAATGCCCC 2400
2401 CTGATGTCGTGTTTAGCTTTTTGCCATTTAGTATATTGTTAGCGAGCTGTTTCAATATATCCAGTTTACATGGCTAATCC 2480
2481 AGTGTTTTGTTTACAGTTATTTTCACCTCCATTGATTTATCGCTCTGTAGACGTTAGCTGACATTACTCTAATAGTTGTG 2560
2561 AAGGAACTTTGTCAACAAATCATTTACATTGTTTATTTAGTTTGGACTCCCACACGGTTGTGTTTGATTCATCCTATGAC 2640
2641 AAAGATAAAGTGACTCACCTTTCTGTGGTGAGCTGACGTCTATTTTCCTGAGGTTAGTAACTGTCCTGAAGGCAATAGCA 2720
2721 TATCTTGCTTTACACATCTTTTTCTTGTGAAAAGGGTCTATGGAAGTCCCTGCATGGTCTAGCTGGCTTGGATGGCCCAA 2800
2801 AAGACAAATCAGAATACCAACAAGAACATCTCCTACTACTCCTTATGGACAGTCTGAGCTTGGAGTATCCCAGTACAAAA 2880
2881 TACTGGGGTGTGGCCAGACACCACAGGCCCCCCTCTACCAATGTGACTCTCTGCCCCCCAATAGTAAAGTCACTGATCTC 2960
2961 ACTCATCTTCAGATCACCATTTTTTCTTTCAAAAAATTACTGATGACGAGCGGAGGACATTTCAGCAAGTCTTGATGTTAG 3040
3041 CTAACAAAGACAAAACAGCTAGAAAAATACAAATATGTAACAATGTTACAAATAACATTAAAGTATTCTTGAGGTTTGTC 3120
3121 TTTATATGTGGTTATGGGTGAAATTCCACTATAGATCAAACACTCTAACGTTTAATGATAACAAAGCCATAAATGCTAAA 3200
3201 TGCTTCTTTTAGCATGTGCTAATCTACACTCCAGGCACTATAGTAAAGATAAGCTTTATTAAAGCTCTAGCAGCTGTGTT 3280
3281 TTCTTTGTCACTGTGAGCTGTTAAAGCATTAAAAATAAAGCTTTTGCTACATTAACCATCAGAGATAATCTCTTGTGTGA 3360
3361 AACTAAATCCTGCATTAGCATTATGCTCTGGAGACTCTTCGGAAAACTTAAGTGCTCTGTTGAAAAGCTCTGGGCTGTGA 3440
3441 CTCAGTCTGAGTAGACGGTATAAAAGCGAATAAAATTAGTTCTTGCTGTTCGGCAAC

**Figure S7. Upstream sequences of piranha *gnrh3,* related to Figure 3 and Figure 4.**

The upstream sequence of piranha *gnrh3,* which was used for generation of transgenic medaka and zebrafish, Tg(*pngnrh3*:GFP).

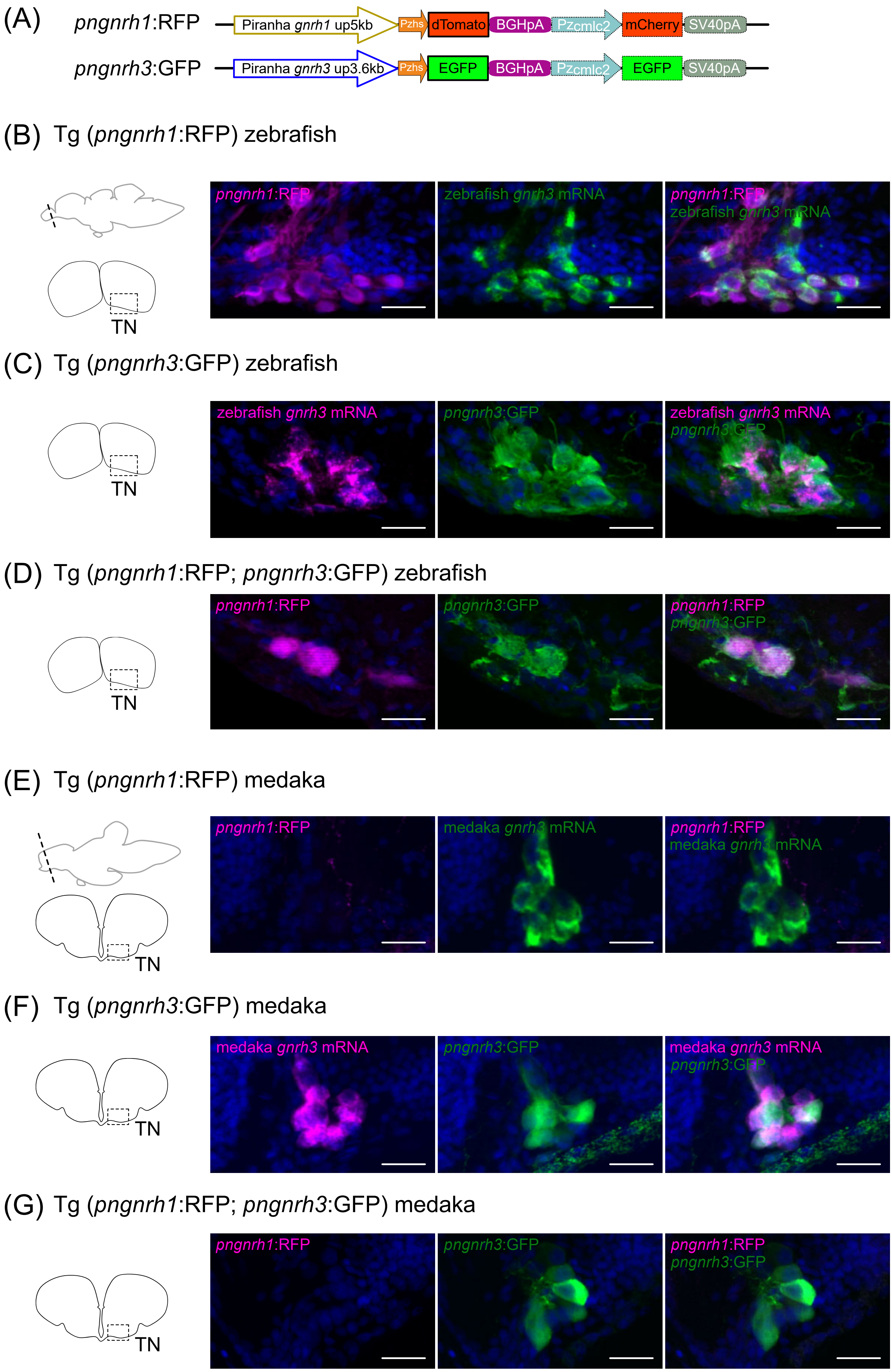

(A)
pngnrh1:RFP
Piranha gnrh1 up5kb
Pzhs
dTomato
BGHpA
Pzcmlc2
mCherry
SV40pA
pngnrh3:GFP
Piranha gnrh3 up3.6kb
Pzhs
EGFP
BGHpA
Pzcmlc2
EGFP
SV40pA
(B) Tg (pngnrh1:RFP) zebrafish
TN
pngnrh1:RFP
zebrafish gnrh3 mRNA
pngnrh1:RFP
zebrafish gnrh3 mRNA
(C) Tg (pngnrh3:GFP) zebrafish
TN
zebrafish gnrh3 mRNA
pngnrh3:GFP
zebrafish gnrh3 mRNA
pngnrh3:GFP
(D) Tg (pngnrh1:RFP; pngnrh3:GFP) zebrafish
TN
pngnrh1:RFP
pngnrh3:GFP
pngnrh1:RFP
pngnrh3:GFP
(E) Tg (pngnrh1:RFP) medaka
TN
pngnrh1:RFP
medaka gnrh3 mRNA
pngnrh1:RFP
medaka gnrh3 mRNA
(F) Tg (pngnrh3:GFP) medaka
TN
medaka gnrh3 mRNA
pngnrh3:GFP
medaka gnrh3 mRNA
pngnrh3:GFP
(G) Tg (pngnrh1:RFP; pngnrh3:GFP) medaka
TN
pngnrh1:RFP
pngnrh3:GFP
pngnrh1:RFP
pngnrh3:GFP

**Figure S8. Examination of enhancer activity of piranha *gnrh1* and *gnrh3* (*pngnrh1*/*pngnrh3*) in the terminal nerve (TN) of zebrafish and medaka, related to Figure 3 and Figure 4.**

(**A**) The constructs used to generate transgenic zebrafish and medaka. Both constructs examine the enhancer activity of piranha *gnrh1* or *gnrh3* 5'flanking regions by using a basal promoter (zebrafish heat shock promoter, Pzhs) and a fluorescent protein (dTomato or EGFP). For screening of embryos, cardiac myosin light chain 2 promoter of zebrafish (Pzcmlc2), mCherry or EGFP and SV40 poly(A) signal were inserted downstream of reporter construct. (**B**) In Tg (*pngnrh1*:RFP) zebrafish, *pngnrh1* enhancer-induced RFP expression is observed in GnRH3 neurons (*gnrh3* mRNA-expressing neurons) in the TN. (**C**) In Tg (*pngnrh3*:GFP) zebrafish, *pngnrh3* enhancer-induced GFP expression is observed in GnRH3 neurons in the TN. (**D**) Analysis of the double transgenic zebrafish, Tg (*pngnrh1*:RFP; *pngnrh3*:GFP). In Tg (*pngnrh1*:RFP; *pngnrh3*:GFP) zebrafish, some of the neurons in the TN express both RFP and GFP suggesting that *pngnrh1* and *pngnrh3* enhancers are activated in the same neurons. (**E**) In Tg (*pngnrh1*:RFP) medaka, *pngnrh1* enhancer-induced RFP expression is not observed in the GnRH3 neurons (*gnrh3* mRNA-expressing neurons) in the TN. (**F**) In Tg (*pngnrh3*:GFP) medaka, *pngnrh3* enhancer-induced GFP expression is observed in the GnRH3 neurons in the TN. (**G**) In double transgenic medaka, Tg (*pngnrh1*:RFP; *pngnrh3*:GFP), some of the neurons in the TN express only GFP suggesting that only *pngnrh3* enhancers are activated in the TN. Scale bars, 100 μm.

**Table S1. The sequence of primers used in this study, related to Figure 1 and Figure 2.**

For full-length cloning of piranha *gnrh1*

5’-CTSYCAGCAYTGGTCITWYGG-3’
5’-ACTGGTCNTWYGGICTIMGICCIGGIG-3’
5’-TACTGCGTGGTCACTTTCAGCTTTCGG-3’
5’-TGCAGCCGCCTGAAAAGTGCCTGTC-3’

For full-length cloning of piranha *gnrh3*

5’-ACCCTSTSYCARCAYTGGTCITAYGGITGG-3’
5’-AGCACTGGTCIYAYGGITGGYWNCCIGGIGG-3’
5’-CARCAYTGGTCITAYGGITGG-3’
5’-TATCTGCTGCAGTGGAGACTCCAGG-3’
5’-GGGGCAAAGCCACAACAGCATCACC-3’

For cloning of head-and-tail-light tetra *gnrh3*

5’-CTSYCAGCAYTGGTCITWYGG-3’
5’-ACTGGTCNTWYGGICTIMGICCIGGIG-3’
5’-CTACTTTAGCGCTTCAGAATCTTCCTCA-3’
5’-TCTCCAGCGTCCATCATTCTGAA-3’
5’-GTGATCTGCTGCAGTGGAGAGTT-3’

For genotyping of piranha *gnrh1* KO

5’-GGCTGTTTTCTGTTGCTCTTTTCAGGATG-3’
5’-TACCGCCTGAAAAGTGCCTGTCA-3’

Supplemental References


S1. Chehade, C., Amaral, F.G., Branco, G.S., Cassel, M., De Jesus, L.W.O., Costa, F.G., Bordin, S.A., Moreira, R.G., and Borella, M.I. (2020). Molecular characterization of different preproGnRHs in *Astyanax altiparanae* (Characiformes): Effects of GnRH on female reproduction. Molecular reproduction and development *87*, 720-734.

S2. Bogerd, J., Ka Wan, L., Janssen-Dommerholt, C., and Goos, H. (1992). Two gonadotropin-releasing hormones from African catfish (*Clarias gariepinus*). Biochemical and biophysical research communications *187*, 127-134.

S3. Zandbergen, M.A., Kah, O., Bogerd, J., Peute, J., and Goos, H.J.T. (1995). Expression and Distribution of Two Gonadotropin-Releasing Hormones in the Catfish Brain. Neuroendocrinology *62*, 571-578.

S4. Torgersen, J., Nourizadeh-Lillabadi, R., Husebye, H., and Aleström, P. (2002). *In silico* and *in situ* characterization of the zebrafish (*Danio rerio*) gnrh3 (sGnRH) gene. BMC Genomics *3*, 25-25.

S5. Steven, C., Lehnen, N., Kight, K., Ijiri, S., Klenke, U., Harris, W.A., and Zohar, Y. (2003). Molecular characterization of the GnRH system in zebrafish (*Danio rerio*): cloning of chicken GnRH-II, adult brain expression patterns and pituitary content of salmon GnRH and chicken GnRH-II. General and comparative endocrinology *133*, 27-37.

S6. Hildahl, J., Sandvik, G.K., Edvardsen, R.B., Fagernes, C., Norberg, B., Haug, T.M., and Weltzien, F.-A. (2011). Identification and gene expression analysis of three GnRH genes in female Atlantic cod during puberty provides insight into GnRH variant gene loss in fish. General and comparative endocrinology *172*, 458-467.

S7. Okubo, K., Amano, M., Yoshiura, Y., Suetake, H., and Aida, K. (2000). A Novel Form of Gonadotropin-Releasing Hormone in the Medaka, *Oryzias latipes*. Biochemical and biophysical research communications *276*, 298-303.

S8. Karigo, T., Kanda, S., Takahashi, A., Abe, H., Okubo, K., and Oka, Y. (2012). Time-of-Day-Dependent Changes in GnRH1 Neuronal Activities and Gonadotropin mRNA Expression in a Daily Spawning Fish, Medaka. Endocrinology *153*, 3394-3404.

S9. Powell, J.F., Zohar, Y., Elizur, A., Park, M., Fischer, W.H., Craig, A.G., Rivier, J.E., Lovejoy, D.A., and Sherwood, N.M. (1994). Three forms of gonadotropin-releasing hormone characterized from brains of one species. Proceedings of the National Academy of Sciences *91*, 12081-12085.

S10. Gothilf, Y., Muñoz-Cueto, J.A., Sagrillo, C.A., Selmanoff, M., Chen, T.T., Kah, O., Elizur, A., and Zohar, Y. (1996). Three Forms of Gonadotropin-Releasing Hormone in a Perciform Fish (*Sparus Aurata*): Complementary Deoxyribonucleic Acid Characterization and Brain Localization. Biology of reproduction *55*, 636-645.
